# Genetic structure, divergence and admixture of Han Chinese, Japanese and Korean populations

**DOI:** 10.1186/s41065-018-0057-5

**Published:** 2018-04-06

**Authors:** Yuchen Wang, Dongsheng Lu, Yeun-Jun Chung, Shuhua Xu

**Affiliations:** 1 Chinese Academy of Sciences (CAS) Key Laboratory of Computational Biology, Max Planck Independent Research Group on Population Genomics, CAS-MPG Partner Institute for Computational Biology (PICB), Shanghai Institutes for Biological Sciences, Chinese Academy of Sciences, Shanghai, 200031 China; 2 University of Chinese Academy of Sciences, Beijing, 100049 China; 3 Integrated Research Center for Genome Polymorphism, Department of Microbiology, The Catholic University Medical College, Seoul, Socho-gu 137-701 South Korea; 4 School of Life Science and Technology ShanghaiTech University, Shanghai, 201210 China; 5 Center for Excellence in Animal Evolution and Genetics, Chinese Academy of Sciences, Kunming, 650223 China; 6 Collaborative Innovation Center of Genetics and Development, Shanghai, 200438 China

**Keywords:** Han Chinese, Japanese, Korean, Genetic ancestry, Population structure, Population divergence, Admixture, SNP

## Abstract

**Background:**

Han Chinese, Japanese and Korean, the three major ethnic groups of East Asia, share many similarities in appearance, language and culture etc., but their genetic relationships, divergence times and subsequent genetic exchanges have not been well studied.

**Results:**

We conducted a genome-wide study and evaluated the population structure of 182 Han Chinese, 90 Japanese and 100 Korean individuals, together with the data of 630 individuals representing 8 populations wordwide. Our analyses revealed that Han Chinese, Japanese and Korean populations have distinct genetic makeup and can be well distinguished based on either the genome wide data or a panel of ancestry informative markers (AIMs). Their genetic structure corresponds well to their geographical distributions, indicating geographical isolation played a critical role in driving population differentiation in East Asia. The most recent common ancestor of the three populations was dated back to 3000 ~ 3600 years ago. Our analyses also revealed substantial admixture within the three populations which occurred subsequent to initial splits, and distinct gene introgression from surrounding populations, of which northern ancestral component is dominant.

**Conclusions:**

These estimations and findings facilitate to understanding population history and mechanism of human genetic diversity in East Asia, and have implications for both evolutionary and medical studies.

**Electronic supplementary material:**

The online version of this article (10.1186/s41065-018-0057-5) contains supplementary material, which is available to authorized users.

## Background

East Asia is one of the world’s most populated places, consisting of about 38% of the Asian population or about 22% of the world-wide population. East Asian peoples, especially the three major ethnicities, Han Chinese, Japanese and Korean, share many similarities in characteristics, for example, yellow skin, black eyes and black hair, short and flat noses, which make them hard to be distinguished by appearance. Moreover, East Asian people use similar languages and words, for example, Chinese characters are shared in Japanese, and also existed in Korean until their recent abolition in the 1940s [1].

While many studies have reported the overall picture of genetic structure of global populations, finer scale details of population structure and relevant issues in East Asia have not yet been well addressed. In fact, in many previous studies, samples of Han Chinese and Japanese populations were generally treated as a single group [2], and notably, Korean samples are frequently absent from many important international collaborative projects due to their assumed similarity to Han Chinese and Japanese samples, such as the International HapMap Project [3] and the 1000 Genomes Project [4]. A recent study [5] provided a landscape of autosomal variation and an overall picture of the genetic relationship of Asian populations. However, fine scale genetic structure and relationship among Han Chinese, Japanese and Korean populations have not been revealed or well-studied, partially due to low density of the data.

Here, we newly genotyped 100 Korean samples, and conducted a joint population genetic analysis with 182 Han Chinese individuals and 90 Japanese individuals. We attempted to address some fundamental questions related to the population histories of the three ethnic groups. First, we asked what the genetic make-up of the three populations is and how well they are differentiated. Second, we attempted to dissect population history of the three ethnic groups and infer their genetic origins based on state-of-art methods developed recently. Finally, we asked whether there were subsequent gene-flow occurred since population splits. We believe these efforts can advance our understanding of human genetic diversity and migration history in East Asia, and provide insightful information for future medical studies in the three East Asian populations.

## Results

### Population structure and genetic relationship

#### Genetic difference measured by F_ST_

To assess the genetic relationship among East Asian groups, we examined genome-wide single nucleotide polymorphism (SNP) data in 1032 individuals representing 12 populations. We first calculated pairwise F_ST_ based on genome-wide SNPs (Additional file 1: Figure S1, Additional file 2: Table S2). The three smallest F_ST_ values are between north Han Chinese (CHB) and south Han Chinese (CHS) (F_ST[CHB-CHS]_ = 0.0014), between Chinese Dai in Xishuangbanna (CDX) and Kinh in Ho Chi Minh City of Vietnam (KHV) (F_ST[CDX-KHV]_ = 0.0024), and between CHB and Korean (KOR) (F_ST[CHB-KOR]_ = 0.0026). Genetic difference between KOR and Japanese (JPT) (F_ST[JPT-KOR]_ = 0.0033) is larger than that between KOR and CHB, but smaller than that between two Mongolian populations (Buryat Mongolian (BMON) and DU Mongolian in Qinghai (QHM) [6], F_ST[BMON-QHM]_ = 0.0053). These results are largely consistent with the geographic distribution of populations. Generally, pairwise F_ST_ between Han Chinese, Japanese and Korean (0.0026~ 0.0090) are greater than that within Han Chinese (0.0014), but are much smaller than that between European (CEU) and any East Asian groups, for example, the smallest F_ST_ between CEU and East Asian populations (F_ST[CEU-BMON]_ = 0.0838; F_ST[CEU-KHV]_ = 0.1059) are higher by an order of magnitude. These results suggested a comparatively closer relationship between Han Chinese, Japanese and Korean. More detailed information about the populations can be found in Additional file 3: Table S1.

#### Phylogenetic trees of populations and individuals

A maximum likelihood (ML) tree reconstructed based on pairwise allele frequency difference provides a better visualization of the genetic relationship of populations (Fig. 2a). All the East Asian populations share a clade, and Mongolians are much closer to European than any other East Asian groups. Among the eight non-Mongolian East Asian populations, the Tibetan population (TIB) shows apparent differentiation from the other seven populations which are comprised of four typical mainland populations (CDX, CHB, CHS and KHV) and three island or peninsula populations (JPRK, JPT and KOR).

An individual level neighbor-joining (NJ) tree of 1002 world-wide individuals based on genotyping differentiation was also constructed (Additional file 4: Figure S2A). African and European individuals cluster together respectively, and East Asians individuals also have their own distinct cluster. In East Asians individuals, Mongolian, Ryukyuan and Tibetan have relatively distinct cluster, while Han Chinese, Japanese and Korean showed a relatively mixed phylogeny, suggest their much closer relationship, although substructures are also apparent. Han Chinese samples also show mixture with Southern populations (CDX and KHV).

Using only 6 populations (two Han Chinese populations, Japanese, Korean and two Mongolian populations) to reconstruct an individual tree, we found the phylogeny of the populations became clearer (Additional file 4: Figure S2B). Japanese individuals have their own cluster and Korean individuals are almost distinct from Han Chinese. North and South Han Chinese mixed together, but still have some substructure.

#### Principal component analysis

**Fig. 1:**
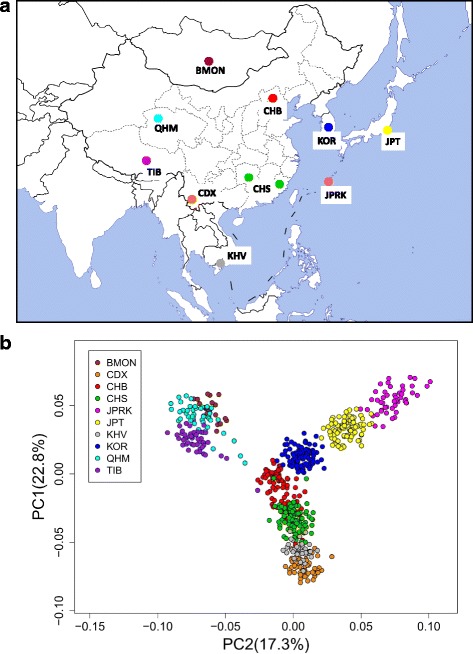
Asian samples location and population relatedness. (**a**) Geographic location of the sampled populations (generated by R 2.15.2 and Microsoft PowerPoint 2010); (**b**) Principal component analysis (PCA) of all the 10 Asian sample populations

**Fig. 2:**
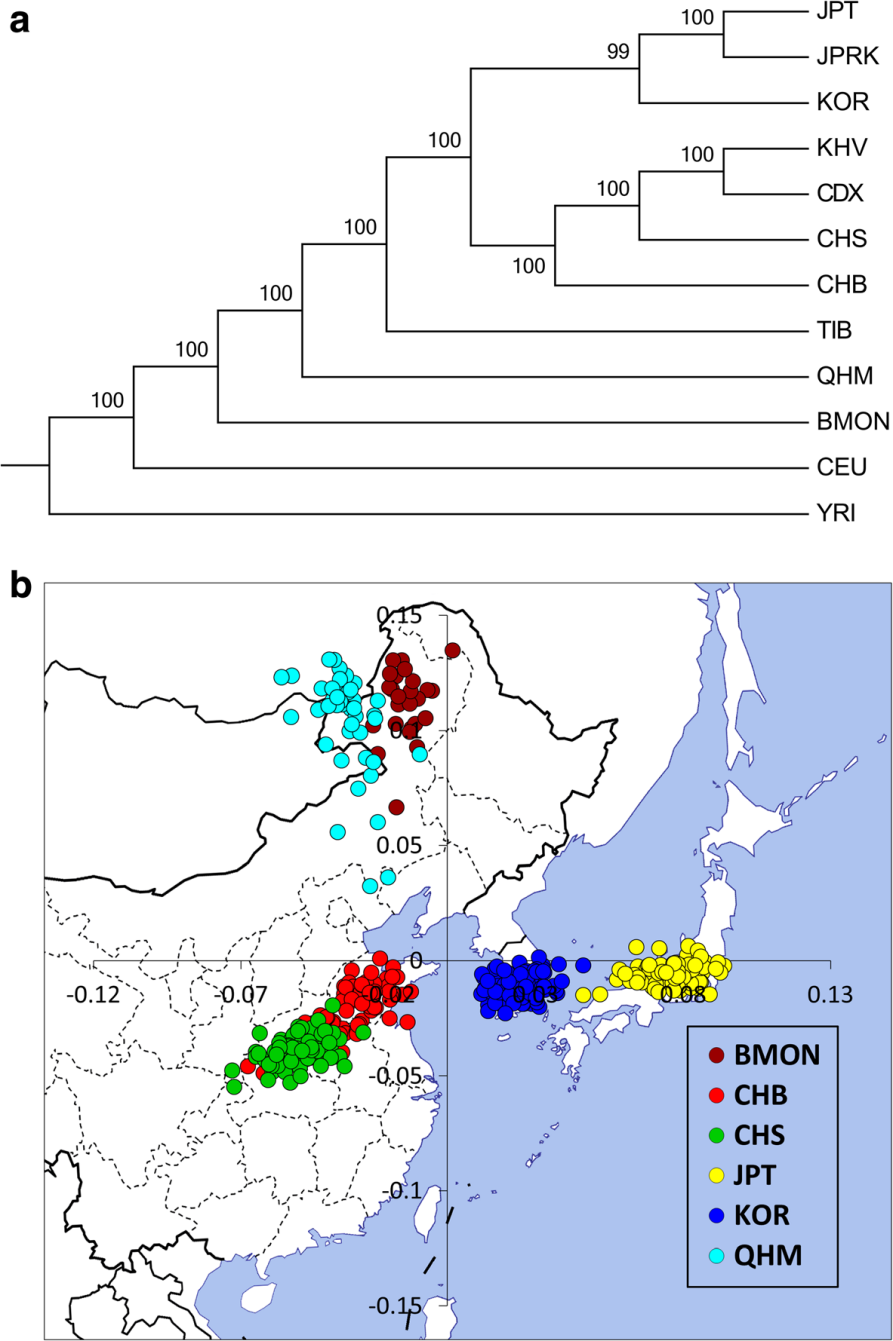
Population level phylogenetic Tree and Principal component analysis (PCA). (A) The maximum likelihood tree was constructed based on pair-wise F_ST_ matrix. And the marked number are bootstrap value; (B) The top two PCs of individuals representing six East Asian populations, mapped to their corresponding geographic locations (generated by R 2.15.2 and Microsoft Excel 2010)

We further performed principal components analysis (PCA) to examine the population structure on individual level (Figs. 1b, 2b, and Additional file 5: Figure S3). We first analyzed East Asian populations with populations worldwide based on 9063 genome-wide SNPs with inter-marker distance > 500 kb (see Methods) (Additional file 5: Figure S3A). The first principal component (PC1) and the second principal component (PC2) explain 9.2 and 4.4% of the total variance (or 58.0% and 28.0% of the top ten PCs), respectively. East Asian groups cluster together closely and are located far from European or African in the PC plot, consistent with their differentiation from other continental populations. We observed some individuals, most of which are Mongolian individuals (BMON or QHM), extend towards CEU cluster, suggesting recent gene flow might have occurred between European and Mongolian populations. Analysis of a dataset with higher density markers (44,549 SNPs, after excluding JPRK samples due to lower marker density, Additional file 5: Figure S3B) showed similar pattern except that individuals were more tightly clustered (PC1 and PC2 explained 10.2% and 4.7% of the variance respectively).

Although East Asian individuals cluster very tightly in the context of analysis of worldwide populations, PCA of ten East Asian populations showed they have substantial substructure (Fig. 1b). For example, Han Chinese (CHB and CHS) and southern populations (CDX and KHV) were separated from other populations by PC1. According to coordinate of PC2 which explained 0.7% of the total variance, Mongolian and Tibetan are closely located in one side, while the island populations (Japanese and Ryukyuan) other side. Except Mongolian and Tibetan, other populations showed much closer relationship in the PC plot (Fig. 1b, Additional file 5: Figure S3C). Some Tibetan and DU Mongolian (QHM) individuals distribute toward CHB cluster, suggesting gene flow between these northern populations. These patterns are even more obvious when JPRK samples were removed from the analysis (Additional file 5: Figure S3C). High density dataset showed more pronounced substructure: On a finer scale, two Mongolian populations also showed substructure, and QHM individuals are sitting between BMON and TIB clusters which is consistent with their history [6]. In all the analyses, Dai (CDX) and Vietnamese (KHV) always cluster closely and can be separated from Han Chinese, Japanese and Korean (Fig. 1b, Additional file 5: Figure S3C).

To examine genetic relationship of the typical East Asian populations, we did a separate PCA with some southern populations excluded (Fig. 2b). PC1 separates Mongolian from the other groups and explain 0.9% of the variance (or 21.9% of the top 10 PCs). PC2 separates populations living in the islands and peninsula from the mainland populations. Japanese and Korean each has each independent cluster, while two Han Chinese populations and two Mongolian both have some overlaps, and some Mongolian individuals are located toward CHB. Interestingly, the distribution of these clusters is generally consistent with the geographical distribution of these populations (Fig. 2b). We next performed PCA of only Han Chinese, Japanese and Korean individuals (Additional file 5: Figure S3D), PC1 explained 0.8% of the variance (or 20.2% of the top 10 PCs) and separated Han Chinese from Japanese and Korean. PC2 separated CHB and KOR from CHS and JPT. Japanese and Korean each has its own cluster and both are located apart from Han Chinese while CHB and CHS cannot be completely separated at this scale. These results suggested that although similar in appearance, Han Chinese, Japanese and Korean are different in terms of genetic make-up, and the difference among the three groups are much larger than that between northern and southern Han Chinese.

#### K-mean clustering analysis

We classified Han Chinese, Japanese and Korean individuals by K-means clustering with first 10 PCs (Additional file 6: Figure S4). Assuming three clusters (K = 3), Han Chinese and Korean are not well distinguished, while all the Japanese individuals cluster perfectly. There are two clusters in Han Chinese and Korean, the north cluster (NC, in orange) and the south cluster (SC, in green). All the Korean individuals belong to NC while CHB and CHS have SC on average of  7.5% and 67.4% of SC, respectively. Assuming four clusters (K = 4), Han Chinese can be distinguished well from Korean. The NC at K = 3 separated into Korean cluster (KC, in blue) and north Han Chinese cluster (NHC, in orange) [7]. Around 64.0% of CHB individuals and 9.0% of KOR individuals are assigned to NHC, while 3.4 and 32.6% of CHB individuals to KC and SC (in green), respectively.

#### Screen ancestry informative markers

We further explored whether the three populations could be well distinguished with a small number of ancestry informative markers (AIMs, see Methods). Individuals from any two populations can be distinguished with a sufficient number of AIMs, and we use Matthews correlation coefficient (MCC, see Methods) to measure the clustering ability of AIMs (Additional file 7: Figure S5). As a result, we could use only 89 SNPs (CHB/KOR), 46 SNPs (CHB/JPT), 44 SNPs (CHS/KOR), 26 SNPs (CHS/JPT) and 73 SNPs (JPT/KOR) respectively to perfectly distinguish each population pairs. These AIMs can facilitate discerning and controlling of population structure among major East Asian populations in future association studies [8].

### Origins, divergence time and migration history of Han Chinese, Japanese and Korean

**Fig. 3:**
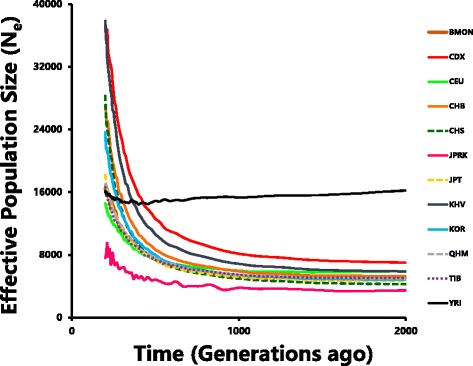
Effective population size varied along time. Effective population size (N_e_) was calculated from LD observations in 2500 distance classes (from 0.001 cM to 2.5 cM with the window size set as 0.001 cM) for each population. Results from 2000 generations ago (GA) to 200 GA are shown (generated by Microsoft Office 365)

To reveal the population history of the three East Asian ethnic groups, we conducted a series of population genetic analysis and estimation. We first estimated the effective population size (N_e_) [9] of East Asian and worldwide populations from 5 to 250 thousand years ago (KYA), assuming 25 years per generation [9] (Fig. 3, Additional file 8: Figure S6). Our analysis showed that African population (YRI) had the greatest N_e_ before 500 generations ago (GA), and N_e_ of European (CEU) was also greater than those of most East Asian populations (except for CDX and KHV) before 1000 GA. However, East Asian populations all showed strong recent growth (~ 1% per generation during the recent 500 generations), so that N_e_ of most East Asian populations exceeded that of YRI during the recent ~ 12.5 KYA. In East Asia, CDX and KHV have the greatest N_e_, followed by CHB. Tibetan (TIB) and Mongolian (BMON and QHM) have greater N_e_ than CHS and KOR before 500 GA, but grew slower than the later ones in recent 7500 years. Because of low marker density of the data, the estimation of N_e_ of JPRK might not be accurate, but apparently N_e_ of JPRK was the smallest. Korean roughly has the same N_e_ with Japanese and two Mongolian populations in history, but showed greater increasing rate in recent 12,500 years.

We further estimated divergence time (T_F_) of each population pair (Tables 1 and 2) based on F_ST_ (Additional file 2: Table S2) and N_e_ following the formula T_F_ = 2N_e_F_ST_ (1) [9]. Most non-African populations diverged from African ~ 82 KYA, consistent with the time of “Out of Africa” as suggested by mtDNA studies [9]. European and East Asian populations diverged later than that between African and non-African populations. Among East Asian populations, CDX diverged from CEU ~ 56 KYA, while the other East Asian populations separated from European about 35~ 50 KYA.

**Table 1:** Pairwise divergence time (T_F_)

T_F_(GA)	BMON	CDX	CEU	CHB	CHS	JPRK	JPT	KHV	KOR	QHM	TIB	YRI
BMON	0	603	1399	276	315	321	265	478	238	88	284	3796
CDX	603	0	2256	218	118	482	374	60	320	1527	509	4518
CEU	1399	2256	0	1839	1690	1369	1676	1985	1782	1896	1589	3290
CHB	276	218	1839	0	24	238	122	149	47	1126	199	3948
CHS	315	118	1690	24	0	238	144	73	81	1059	236	3682
JPRK	321	482	1369	238	238	0	79	379	179	894	329	3156
JPT	265	374	1676	122	144	79	0	285	54	1030	279	3685
KHV	478	60	1985	149	73	379	285	0	235	1347	409	4172
KOR	238	320	1782	47	81	179	54	235	0	1050	225	3826
QHM	88	1527	1896	1126	1059	894	1030	1347	1050	0	998	3988
TIB	284	509	1589	199	236	329	279	409	225	998	0	3592
YRI	3796	4518	3290	3948	3682	3156	3685	4172	3826	3988	3592	0

Note: Pairwise divergence time (T_F_), in units of generations ago (GA), estimated by 2N_e_F_ST_

**Table 2:** Results of F_4_ test

popO	popA	popB	popC	F_4_(A,O;C,B)	F_4_(B,O;C,A)
YRI	TIB	CDX	CHB	0.01255	0.01977
YRI	TIB	CDX	CHS	0.01019	0.02478
YRI	TIB	CDX	KOR	0.01333	0.01780
CEU	TIB	CDX	CHB	0.01063	0.02007
CEU	TIB	CDX	CHS	0.00957	0.02605
CEU	TIB	CDX	KOR	0.01238	0.01917
YRI	CDX	JPRK	CHB	0.01317	0.00911
YRI	CDX	JPRK	CHS	0.01818	0.00843
YRI	CDX	JPRK	JPT	0.00723	0.02230
YRI	CDX	JPRK	KOR	0.01119	0.01547
CEU	CDX	JPRK	CHB	0.01239	0.00734
CEU	CDX	JPRK	CHS	0.01838	0.00788
CEU	CDX	JPRK	JPT	0.00728	0.02052
CEU	CDX	JPRK	KOR	0.01150	0.01442
YRI	TIB	JPRK	JPT	0.00766	0.02798
YRI	TIB	JPRK	KOR	0.01241	0.02116
CEU	TIB	JPRK	JPT	0.00769	0.02849
CEU	TIB	JPRK	KOR	0.01267	0.02239

Note: population A (popA) and population B (popB) are two donate populations, population C (popC) is a target population who received gene flow, population O (popO) is an out group. Additional file 14: Figure S10C shows the model of F_4_ test. The value of F_4_(A,O;C,B) stands for the size of the common part of the two pathway A to O and C to B (labeled as l) on the tree weighted by the gene flow proportion α (labeled as αl), and similarly F4(B,O;C,A) equals (1-α)m. And the calculation of F_4_ value noted in METHODS

We found that the present-day Han Chinese and Japanese have the most recent common ancestor that can be dated back about 3.0~ 3.6 KYA (corresponding to the Shang Dynasty in Chinese history). Korean and northern Han Chinese had frequent communications in ancient time, and the divergence time between the two populations was estimated as ~ 1.2 KYA (corresponding to the later period of Three Kingdoms of Korea, or the Tang Dynasty in China). And Japanese and Korean separated ~ 1.4 KYA, a little earlier than that of Han Chinese and Korean (corresponding to Asuka period in Japan, or in the middle of Three Kingdoms period of Korea).

### Gene flow among east Asian populations

Considering their frequent communication in history, we are curious about how the initial common origin and recent gene flow contributed to the genetic make-up of Han Chinese, Japanese and Korean populations.

#### STRUCTURE and ADMIXTURE analysis

To examine fine-scale population structure and assess the genetic make-up of East Asian groups, we applied a model-based method, *STRUCTURE* [10], to analyze the genome-wide data with that of worldwide populations. East Asian populations showed distinct genetic component inferred from STRUCTURE which is totally different from those of European or African (Additional file 9: Figure S7). There is no more than 0.5% of African’s contribution in East Asian’s genome can be detected, while in Mongolian there is considerable contribution from European ancestry (12.5~ 16.9%) (Additional file 9: Figure S7, K = 3). We identified a component which we named northern East Asian component (NEAC) existing widely in the East Asian populations (Additional file 9: Figure S7, in red). On the contrary, southern East Asian component (SEAC) exists mainly in typical mainland populations (CDX, CHB, CHS and KHV) (Additional file 9: Figure S7, in green), and Ryukyuan component (RC) exists mainly in island and peninsula populations (JPRK, JPT and KOR) (Additional file 9: Figure S7, K = 5~ 7, in yellow). QHM has some Tibetan component (TC) (~ 8.98%) while BMON has very limited TC (~ 0.5%) (Additional file 9: Figure S7, K = 7, in purple). We could also observe considerable TC contribution in CHB and JPRK (5.2 and 2.6%, respectively) (Additional file 9: Figure S7, K = 7, in purple).

To further assess finer scale population structure and genetic composition of East Asian populations, we run independent STRUCTURE analyses in the ten East Asian populations without non-Asian population samples (Additional file 10: Figure S8). At K = 3, we observed a clear north/south cline of genetic components. SEAC (in green) constituted the majority (92.7%) of the CDX genome; Mongolian and Tibetan showed distinct composition but both were influenced by NEAC significantly (5.7%~ 15.0%) (Additional file 10: Figure S8, K = 4, in red). At K = 5, Mongolian had different composition from Tibetan but showed considerable contribution from the Tibetan component (TC) (3.1~ 14.5%), especially in QHM, who migrated to Qinghai-Tibetan Plateau about 500 years ago and were reported to have adapted to local environment [6]. TC was also observed widely in other populations such as Han Chinese and Korean (2.3~ 12.6%) (Additional file 10: Figure S8, K = 5, in purple). At K = 6, RC was separated into two different components, one (in pink) mainly exists in island populations (JPT and JPRK) and has very little (1.4~ 1.7%) contribution to other populations, and the other (in yellow) exists both in island populations (JPRK and JPT, 39.1~ 66.7%) and northern populations (BMON, CHB and KOR, 4.3~ 41.7%) (Additional file 10: Figure S8, K = 6).

**Fig. 4:**
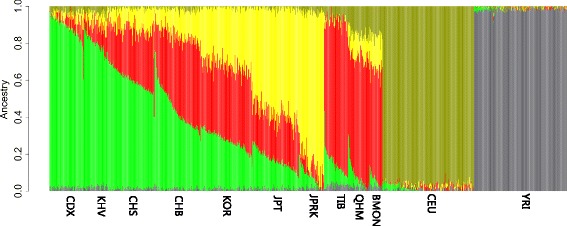
Admixture analysis of East Asian populations together with world-wide non-Asian populations. Result of K = 5 was shown and each vertical bar represents genome makeup of an sample individual (calculated by ADMIXTURE 1.23 [1] and R 2.15.2)

We confirmed the ancestry inference results with another commonly used method, *ADMIXTURE* [11] (Fig. 4). We ran ADMIXTURE from K = 2 to K = 12 and chose results at K = 5 as the best inference according to *ADMIXTURE*’s cross-validation procedure (the best K has the lowest cross-validation error) (Additional file 11: Figure S9). Similar to the STRUCTURE results, East Asian individuals demonstrated three typical ancestry components which were entirely different from African or European. No strong influence of African ancestry on East Asian populations was detected (3.1% in TIB and no more than 2.4% in any other East Asian populations). However, we observed considerable presence of European ancestry in Mongolian population (15.5~ 16.1%) and some other East Asian populations (up to 6.4%). As shown in Fig. 4, the three dominant ancestral components in East Asian populations are NEAC (in red), SEAC (in green) and RC (in yellow). NEAC is also dominant in Tibetan and Mongolian and uniformly distributes in Han Chinese (27.3 to 39.4%), Japanese (24.5%) and Korean (39.9%). SEAC and RC are frequent in CDX/KHV and JPRK respectively, and both exist in other East Asian populations, while their proportions are generally negatively correlated across populations.

#### F test and D test

We used the F_3_ test [12, 13] to detect potential admixture populations and a significant negative F_3_ value suggests existence of gene flow and possible admixture pattern. We tried each populations combination (F_3_ (population C; population A, population B)) and listed those with negative F_3_ values (Additional file 12: Table S3). We found that to some degree Han Chinese, Japanese, Korean populations each received gene flow from the other two. According to the F_3_ results, Han Chinese may have common origin with CDX and KHV, and receive gene flow from northern groups (NEAC) and from islands groups (RC). Japanese has significant JPRK contribution and can be treated as an admixture of Ryukyuan and other East Asian populations (Additional file 12: Table S3). The Korean population also has NEAC, SEAC and RC, but F_3_ results strongly suggest that it received gene flow directly through Han Chinese and Japanese (Additional file 12: Table S3). Our results showed that Mongolian populations (BMON and QHM) were admixed by European and some East Asian populations (for example, Han Chinese or Japanese, or their common ancestry, Additional file 12: Table S3), consistent with their known history.

We further performed F_4_ test [12, 13] to quantitatively estimate the gene flow from surrounding groups to Han Chinese, Japanese and Korean populations, respectively. F_4_(A, B; C, D) denotes the common part of two drift route A to B and C to D (Additional file 13: Figure S11C). If A and B are two reference populations, C is a target population and O is the outgroup, then F_4_(O, A; B, C) and F_4_(O, B; A, C) have linear relation, which is equal to the gene flow ratio multiplied by common drift route (αl and (1-α)m, Additional file 13: Figure S11C). Therefore, we can use linear regression method to estimate the gene flow (Additional file 14: Figure S10). As a result, NEAC contributed 52.2% to CHB, 44.0% to CHS, 19.7% to JPT and 37.5% to KOR; RC contributed 14.3% to CHB, 12.4% to CHS, 52.6% to JPT and 33.8% to KOR; SEAC contributed 33.5% to CHB, 43.6% to CHS, 27.7% to JPT and 28.7% to KOR.

Finally, we applied the D test (4 population test) [12] to qualitatively estimate the gene flow Between Han Chinese, Japanese and Korean populations (Table 3). In D test, a significant nonzero D values suggest the existence of gene flow (Additional file 13: Figure S11, more information can be found in Material and Methods part). Significant negative values were observed when D test were applied for comparing gene flow between Han Chinese and Japanese/Korean, indicating greater gene flow between Han Chinese and Korean than that between Han Chinese and Japanese. We further applied D test for detecting gene flow between Japanese/Korean and south/north Han Chinese, the D values were not significantly different from 0, indicating the gene flow between north Han Chinese and Japanese/Korean are almost equal to that between south Han Chinese and Japanese/Korean. These results remain consistent when different outgroups (CEU, YRI) were used in the analysis (Table 3).

**Table 3:** Results of D test

popW	popA	popY	popZ	D value	Z score
CHB	YRI	JPT	KOR	−0.0055	−5.061
CHS	YRI	JPT	KOR	−0.0054	−4.865
JPT	YRI	CHB	CHS	0.0014	1.515
KOR	YRI	CHB	CHS	0.0016	1.649
CHB	CEU	JPT	KOR	−0.0062	−6.748
CHS	CEU	JPT	KOR	−0.0060	−6.596
JPT	CEU	CHB	CHS	−0.0005	−0.595
KOR	CEU	CHB	CHS	−0.0004	−0.446

Note: Population A (popA) is outgroup, population W (popW) is a reference population, population Y (popY) and population Z (popZ) are two potential target populations. If D value is significantly positive, it suggests that the gene flow between population W and population Y was greater than that between population W and population Z. On the contrary, if D value is significantly negative, it means the gene flow between population W and population Y was smaller than that between population W and population Z. If D value is not significantly positive or negative, it suggests that the gene flow between population W and population Y are equal to that between population W and population Z, or there is no gene flow between population W and population Y or between population W and population Z

## Discussion

In this project, we conducted a genome-wide study of three East Asian populations based on genome-wide high-density SNP data. Our results showed that the three East Asian populations, Han Chinese, Japanese and Korean, although they resemble each other in appearance, have distinguishing genetic make-up and are differentiated apparently on genomic level. In this respect, we suggest the three East Asian ethnic groups should be treated as independent populations in the future studies rather than as a single group, particularly in evolutionary studies or medical studies where population structure matters. In addition to global differentiation, the AIMs we screened also indicated considerable differentiation of local genomic regions among the three populations. For example, several highly differentiated SNPs (AIMs) are enriched in the *CD46* gene (on chromosome 1q32), which is a type I membrane protein and is a regulatory part of the complement system [14, 15]. These highly differentiated genomic regions could be due to regional adaptation to some pathogens, although further studies are necessary to validate these signals and confirm this conclusion. These results further suggest the importance to consider population difference when medical studies are conducted in the three East Asian ethnic groups.

It is obvious that the genetic difference among the three East Asian groups initially resulted from population divergence due to pre-historical or historical migrations. Subsequently, different geographical locations where the three populations are located, mainland of China, Korean Peninsular and Japanese archipelago, respectively, apparently facilitated population differentiation due to physical isolation and independent genetic drift. Our estimations of population divergence time among the three groups, 1.2~ 3.6 KYA, are largely consistent with known history of the three populations and those related. However, considering that recent admixture could have reduced genetic difference between populations, it is likely the divergence time was underestimated.

We detected substantial gene flow among the three populations and also from the surrounding populations. For example, based on our analysis with the F_3_ test, Korean received gene flow from Han Chinese and Japanese, and gene flow also happened between Han Chinese and Japanese (Additional file 12: Table S3). These gene flows are expected to have reduced the genetic differentiation between the three ethnic groups. On the other hand, we also detected considerable gene flow from surrounding populations to the three populations studied. For instance, an ancestral population represented by Ryukyuan have contributed greater to Japanese than to Han Chinese, while southern ethnic group like Dai have contributed more to continent populations than to island and peninsula populations. Contrary to the gene flow among the three populations, these gene flows from surrounding populations are expected to have increased genetic difference among the three populations if they occurred independently and from different source populations. According to our results, the major source of gene flow to the three ethnic groups were substantially different, for example, the major source of gene flow to Han Chinese was from southern ethnic groups, the major source of gene flow to Japanese was from southern islands, and the major source of gene flow to Korean were from both mainland and islands. Therefore, those gene flows might have significantly contributed to further genetic differentiation of the three populations.

The three populations have similar but not identical demographical history; they all experience a strong population expansion in the last 20,000 years. However, according to different geographic distribution, their effective population size and population expansion are different. North Han Chinese has greater N_e_ than south Han Chinese in early time. Japanese and Korean have similar N_e_ in most of the history, while Korean population expanded much faster than Japanese in recent thousands of years, suggested a higher potential for population expansion in peninsula than in islands. Our estimation showed that Han Chinese have much greater expansion speed than Japanese and Korean, suggested continental environment has even higher potential for population expansion than peninsula or islands. However, the estimated N_e_ increase in East Asian populations could be, at least partially, explained by recent population admixture which is expected to increase population genetic diversity thus increase N_e_.

Finally, as we briefly mentioned above, natural selection might have substantially contributed to the genetic diversity of the populations. We identified several genomic regions with significant differentiation between Han Chinese, Japanese and Korean. For example, most highly differentiated SNPs enriched in the genes that related to neurological system process and cell-cell signal transmission. There are also significant selective signals in detected in EGF-like domain and Collagen-like domain. The physiological significance and evolutionary importance of these selective signals needed more study.

## Conclusion

In summary, the genetic structures of the present-day Han Chinese, Japanese and Korean people were shaped jointly by initial population divergence, geographical isolation, subsequent gene flow and possibly regional natural selection. Our analysis of the genome-wide data of these populations and their surrounding neighbors should advance our understanding of population history and mechanism of human genetic diversity in East Asia.

## Methods

### Population and samples

Peripheral blood samples of 100 Koreans unrelated individuals were collected from South Korea. Each individual was the offspring of a non-consanguineous marriage of members of the same nationality within three generations. Informed consents were acquired from the participants. All procedures followed were in accordance with the ethical standards of the Responsible Committee on Human Experimentation (approved by the Biomedical Research Ethics Committee of the Shanghai Institutes for Biological Sciences, No. ER-SIBS-261408) and the Helsinki Declaration of 1975, as revised in 2000. In addition, genome-wide single nucleotide polymorphisms (SNP) data of 182 unrelated Han Chinese, and 90 Japanese individuals were collected from the HapMap Project (International HapMap, 2003) and the 1000 Genomes Project [4], and comparisons were made with 663 individuals (630 individuals through Quality Control) representing 8 populations worldwide from the HapMap Project, the 1000 Genomes Project and the PanAsia SNP Project [5] (Fig. 1a, Additional file 3: Table S1).

### Genotyping and data quality control

A set of 934,968 loci were genotyped in 100 Korean samples with Affymetrix Genome-wide human SNP Array 6.0. *.CEL files containing raw intensity data were analyzed with Birdsuite version 1.5.3 [16]. Only autosomal SNPs with missing rate less than 0.05 were employed for the downstream analyses. For the purposes of this study, only SNPs with Reference Sequence (RS) numbers and vendor-specified strands were used in combining data. For different purposes of analysis, several combined data sets were generated. The first two data sets (dataset 1 and dataset 2) contains 13,576 SNPs for 1002 individuals from all the twelve populations and 733,113 SNPs for 439 individuals from six East Asia populations (BMON, CHB, CHD, JPT, KOR and QHM). These datasets were used to estimate ASD. And dataset 1 was also prepared for principal component analysis [17], STRUCTURE [10] and Admixture [11] analyses, and estimation of gene flow, so that SNPs in high linkage disequilibrium (LD) have been removed from this data set. The third dataset (dataset 3) contains 44,549 SNPs for 596 individuals representing nine East Asian populations (except JPRK samples), which was used for population structure analyses of East Asian populations only. In the fourth dataset (dataset 4), we kept all the SNPs genotyped in all the samples respectively, and used for estimating F_ST_ and screening AIMs [8]. The last dataset (dataset 5) was based on the dataset 4 but phased particularly for calculating LD.

To control for potential batch effect during our data integration, we corrected allele frequency of BMON and QHM [6] by comparing genotypes of the identical CHB and JPT individuals between the data set of [6] and our own data. Because CHB and JPT were also included in the same data set together with BMON and QHM in [6]. To avoid any possible systematic bias introduced during data integration, we did SNP re-calling for HapMap individuals from raw Affymetrix intensity data (.CEL files).

### Population structure analysis

Genetic difference between populations was measured by F_ST_ which was calculated following an unbiased estimate [18]. Principal component analysis (PCA) was performed at individual level using EIGENSOFT [17]. The map was drawn by R (version 2.15.2) based on the information provided by http://www.mapsofworld.com/. We used an allele sharing distance (ASD) as a measure of genetic distance between individuals, and a 1002 × 1002 and a 439 × 439 inter-individual genetic distance matrixes were generated according to genotypes of 13,576 and 733,113 autosomal SNPs respectively (dataset 1&2). The tree of individuals was reconstructed based on ASD distance and using Neighbor-Joining algorithm [19] with the Molecular Evolutionary Genetics Analysis software package (MEGA version 4.0) [20]. Trees of populations as well as components were reconstructed using maximum likelihood method [21] with CONTML program in PHYLIP package (version 3.695) [22]. K-mean clustering analysis was done with R software. Ancestry of each person was inferred using a Bayesian cluster analysis as implemented in the STRUCTURE program [23, 10]. We ran STRUCTURE from *K* = 2 to *K* = 7 and repeated 5 times for each single *K*. All STRUCTURE runs used 15,000 iterations after a burn-in of length 15,000, with the admixture model and assuming that allele frequencies were correlated [23]. Admixture [11] was also employed for the same purpose.

We screened Ancestry Informative Markers (AIMs) [8] to distinguish Han Chinese, Japanese and Korean. These AIMs were selected by the following three criteria: 1) allele frequency highly differentiated between each two populations (measured by F_ST_); 2) inter-marker distance larger than 500 Kb; 3) Matthews correlation coefficient (MCC) of a panel of AIMs based on PC1 not less than 1.0. MCC is defined as the follows:

5 $$ \mathrm{MCC}=\frac{TP\times TN- FP\times FN}{\sqrt{\left( TP+ FP\right)\left( TP+ FN\right)\left( TN+ FP\right)\left( TN+ FN\right)}} $$

where TP is the number of true positives, TN is the number of true negatives, FP is the number of false positives and FN is the number of false negatives [8]).

### Estimation of effective population size and divergence time

We first phased the genotype data with BEAGLE [24]. In order to improve the accuracy of the haplotype inference (phasing), we combined those datasets from the same source and did further estimation independently.

Then we calculated pairwise linkage disequilibrium (r^2^) of each SNPs pair with genetic distance less than 0.25 cM in each population. And effective population size (N_e_) of *t* generations ago (*t* = 1/2*c* (2) [9]) can be estimated for each population in each distance category as N_e_ = 1/(4*c*)*[(1/r_LD_^2^)-2] (3) [9], where *c* is the genetic distance. All r_LD_^2^ are adjust as (r_LD_^2^–1/n), where n is sample size (number of chromosomes) [9]. Divergence time (T_F_) can be estimated by 2N_e_F_ST_ (4) [9]. Here, F_ST_ values were still calculated following an unbiased estimate. N_e_ was calculated as the average of the harmonic means of the relevant recombination distance categories. We used distances categories 0.01 cM to 0.25 cM (corresponding to 200 to 5000 generations ago) to estimate inter-population N_e_ [9].

### Gene flow estimation

We used D test and F test to detect and estimate gene flow [12]. All the variations and Z scores are calculated by delete-m jackknife [25].

In D test, D value indicates the difference of the two allele patterns ABBA and BABA (Additional file 13: Figure S11A). A significant positive or negative D value (|Z| > 2.58, *p* < 0.01) suggests the existence of gene flow either from W to Y or from W to Z. X is out group. In population genetics, D is calculated by

6 $$ \widehat{N}{um}_i/\widehat{D}{en}_i $$

where

7 $$ \widehat{N}{um}_i=\left(w-x\right)\left(y-z\right) $$

8 $$ \widehat{D}{en}_i=\left(w+x-2 wx\right)\left(y+z-2 yz\right) $$

w, y, x and z are sample allele frequency of population W, Y, X, Z.

In F test, F_2_ value is calculated by

9 $$ {F}_2\left(A,B\right)=E\left[{\left({a}^{\hbox{'}}-{b}^{\hbox{'}}\right)}^2\right] $$

(a and b are sample allele frequency of population A and B) and can be looked as the drift on edge A to B in a phylogeny tree. And

10 $$ {F}_3\left(C:A,B\right)=E\left[\left({c}^{\hbox{'}}-{a}^{\hbox{'}}\right)\left({c}^{\hbox{'}}-{b}^{\hbox{'}}\right)\right] $$

denotes the drift on the common path of edge C to A and C to B (D_x_, Additional file 13: Figure S11B). If there is no admixture, F_3_ value should be positive. A significant negative F_3_ value suggests the existence of gene flow [26, 12, 13]. Similarly,

11 $$ {F}_4\left(A,B:C,D\right)=E\left[\left({a}^{\hbox{'}}-{b}^{\hbox{'}}\right)\left({c}^{\hbox{'}}-{d}^{\hbox{'}}\right)\right] $$

If there was gene flow in history, the drift from one population to another would go through more than one path in the phylogeny tree; the ratio of drift along each path is decided by the ratio of gene flow. If there were gene flow from population A to population C (with a ratio α) and from population B to population C (with a ratio 1-α), then

12 $$ {F}_4\left(A,O;C,B\right)=\alpha l $$

13 $$ {F}_4\left(B,O;C,A\right)=\left(1-\alpha \right)m $$

Where *l* and *m* are unknown, but if there is a series of population C that are admixed with ancestry from both population A and population B (A’ and B′) with different ratio, we can use a linear regression to eliminate these parameters, so that we can calculation each gene flow ratio α [13].

## Additional files

Additional file 1: **Figure S1.** A heat map of pair-wise F_ST_. A warmer (red) color means a larger F_ST_ value (generated by R 2.15.2). (PDF 6 kb)

Additional file 2: **Table S2.** Global F_ST_ values between East Asian populations and world-wide groups. (DOCX 16 kb)

Additional file 3: **Table S1.** Genotype data used in this research. (DOCX 21 kb)

Additional file 4: **Figure S2.** Individual level Neighbor-Joining Tree. (A) The NJ tree was constructed according pair-wise genotyping difference; (B) Individual NJ Tree of Han Chinese, Japanese, Korean and Mongolian individuals (generated by MEGA version 4.0). (PDF 6971 kb)

Additional file 5: **Figure S3.** Principle component analysis (PCA). (A~B) PCA results of East Asian groups with CEU and YRI. (C) PCA result of groups within East Asia populations, excluding JPRK individuals for a higher marker density. (D) PCA result of four East Asian populations, including Han Chinese, Japanese and Koreans samples. (PDF 2458 kb)

Additional file 6: **Figure S4.** K-means Cluster of PCA results. The results of Figure S3D were used to conduct the K-means cluster. Individuals that cluster together according to the top 10 PCs would be painted by the same color. Results of K = 3 and K = 4 are shown (generated by R 2.15.2). (PDF 116 kb)

Additional file 7: **Figure S5.** Matthews correlation coefficient (MCC) values increase when more AIMs are used to distinguish individuals. X axis is number of SNPs and Y axis is MCC values. (A) CHB-CHS; (B) CHB-JPT; (C) CHB-KOR; (D) CHS-JPT; (E) CHS-KOR; (F) JPT-KOR (generated by Microsoft Excel 2010). (PDF 99 kb)

Additional file 8: **Figure S6.** Effective population size varied along time. Results from 10,000 generations ago (GA) to 200 GA are shown (generated by Microsoft Office 2010). (PDF 112 kb)

Additional file 9: **Figure S7.** STRUCTURE analysis of East Asian samples with worldwide populations. Results from K = 3 to K = 7 are shown. Each vertical bar represents an individual and each color stands for a genetic component (generated by R 2.15.2). (PDF 141 kb)

Additional file 10: **Figure S8.** STRUCTURE Analysis of ten East Asian populations. Results from K = 2 to K = 6 are shown. Each vertical bar represents an individual and each color stands for a genetic component (generated by R 2.15.2). (PDF 152 kb)

Additional file 11: **Figure S9.** Cross-validation (CV) plot for the Admixture analysis. Result of K = 3 to K = 7 are shown. (PDF 158 kb)

Additional file 12: **Table S3.** Part of F_3_ test results. (DOCX 18 kb)

Additional file 13: **Figure S11.** Models used in gene flow study. (A) Model of D test. X is an out group, we put CEU or YRI in X; W is the contributor while Y and Z are the receivers. By comparing the two allelic patterns BABA and ABBA, we can infer the relationship of scale of gene flow between W to Y and W to Z. (PDF 30 kb)

Additional file 14: **Figure S10.** Results of linear regression in F_4_ test. For each group (Outgroup O, donate population A, donate population B, target population X), X axis is F_4_(B, O; X, A), Y axis is F_4_(A, O; X, B). (A) Outgroup is YRI or CEU, donate populations are TIB and CDX, target population is Han Chinese or KOR; (B) Outgroup is YRI or CEU, donate populations are CDX and JPRK, target population is Han Chinese or JPT or KOR; (C) Outgroup is YRI or CEU, donate populations are TIB and JPRK, target population is JPT or KOR (generated by Microsoft Excel 2010). (PDF 226 kb)

## Abbreviations

AIMs: Ancestry informative markers
ASD: Allele sharing distance
BMON: Buryat Mongolian
CDX: Chinese Dai in Xishuangbanna
CEU: Northern Europeans from Utah
CHB: North Han Chinese
CHS: South Han Chinese
IBD: Isolation by distance
JPRK: Ryukyuan
JPT: Japanese in Tokyo
KHV: Kinh in Ho Chi Minh City, Vietnam
KOR: South Korean
LD: Linkage disequilibrium
MCC: Matthews correlation coefficient
ML tree: Maximum likelihood tree
NEAC: Northern East Asian component
NJ tree: Neighbor-joining tree
PCA: Principal components analysis
QHM: Deedu (DU) Mongolians in Qinghai-Tibetan Plateau
RC: Ryukyuan component
RS: Reference Sequence
SEAC: Southern East Asian component
TC: Tibetan component
TIB: Tibetan
YRI: Yoruba in Ibadan, Nigeria
