# Supplementary figures and images for: Genetic structure, divergence and admixture of Han Chinese, Japanese and Korean populations
Source: Hereditas. 2018 Apr 6;155:19. doi: 10.1186/s41065-018-0057-5 (PMC5889524; doi:10.1186/s41065-018-0057-5)

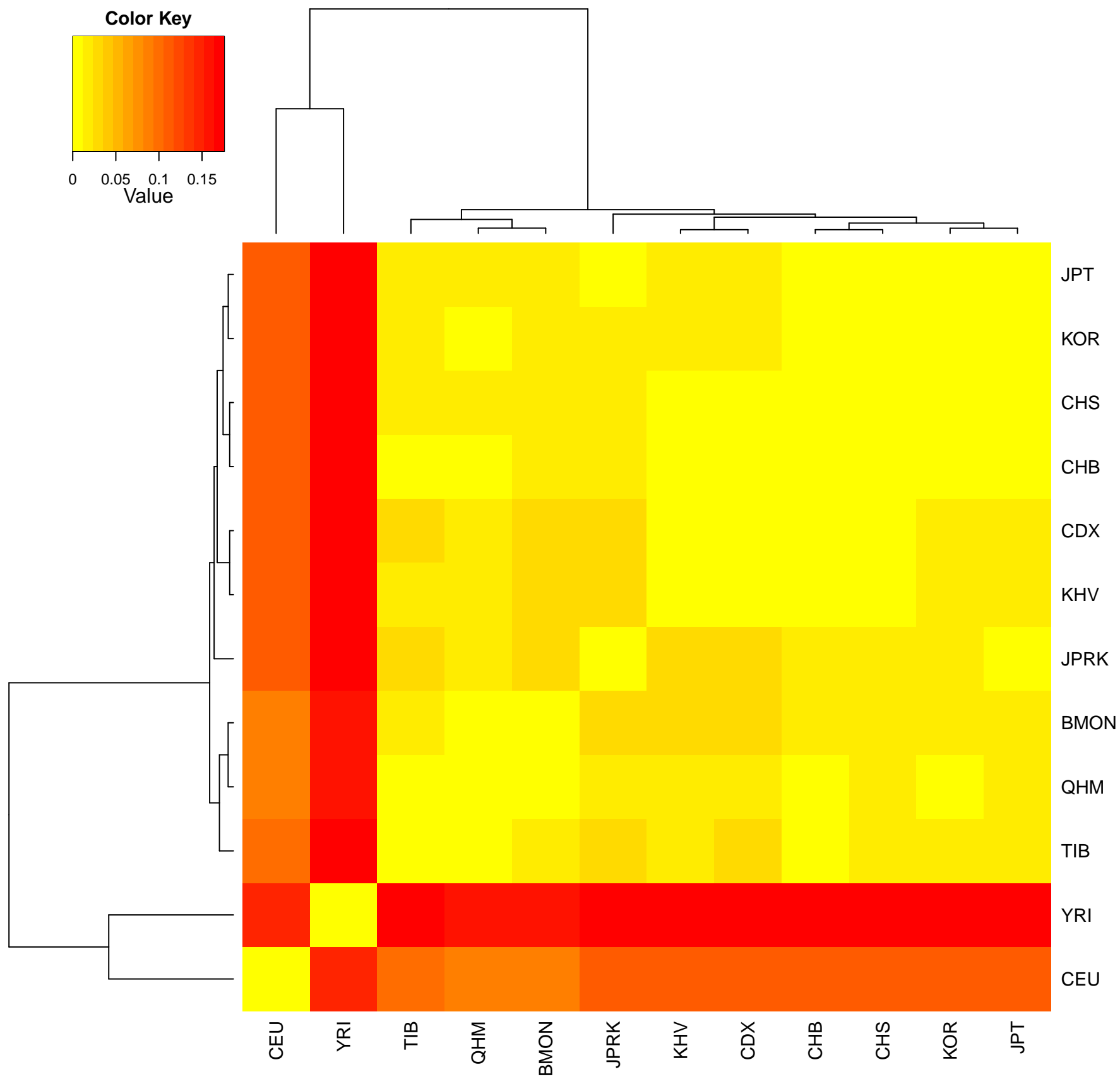

Supplement: Supplementary file 1 — Figure S1. A heat map of pair-wise FST. A warmer (red) color means a larger FST value (generated by R 2.15.2). (PDF 6 kb) [file 41065_2018_57_MOESM1_ESM.pdf]

A

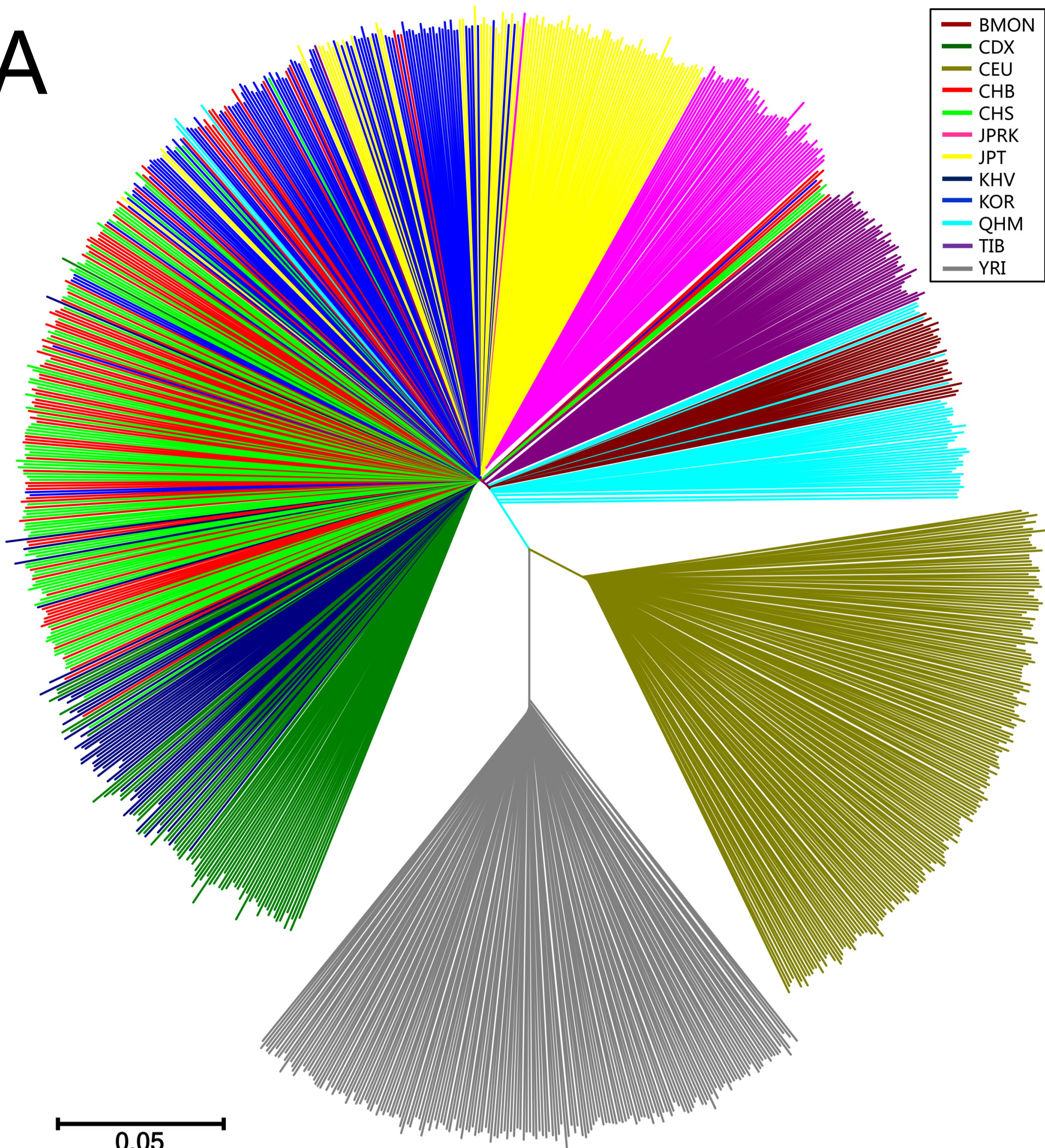

B

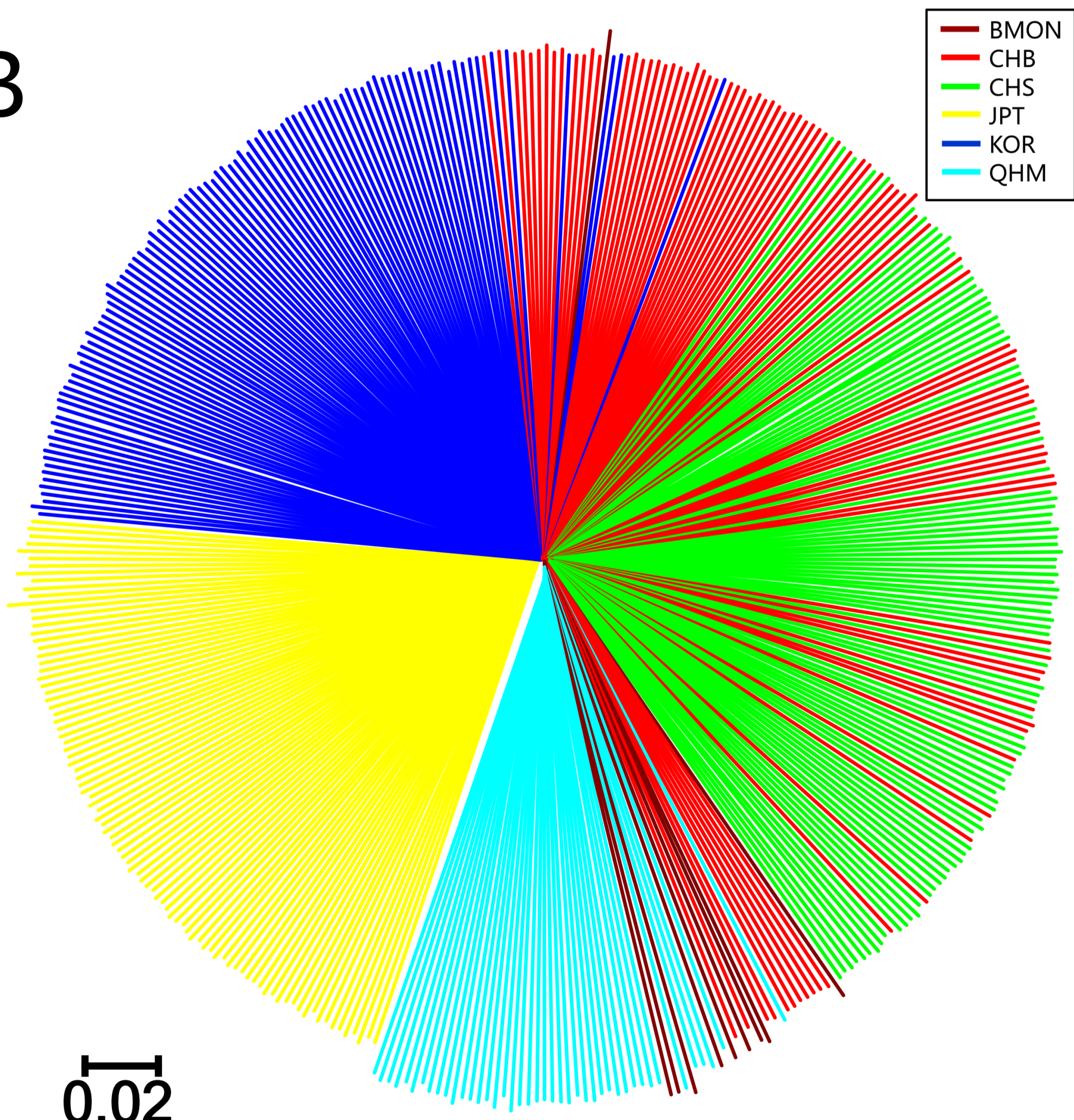

Supplement: Supplementary file 4 — Figure S2. Individual level Neighbor-Joining Tree. (A) The NJ tree was constructed according pair-wise genotyping difference; (B) Individual NJ Tree of Han Chinese, Japanese, Korean and Mongolian individuals (generated by MEGA version 4.0). (PDF 6971 kb) [file 41065_2018_57_MOESM4_ESM.pdf]

**A**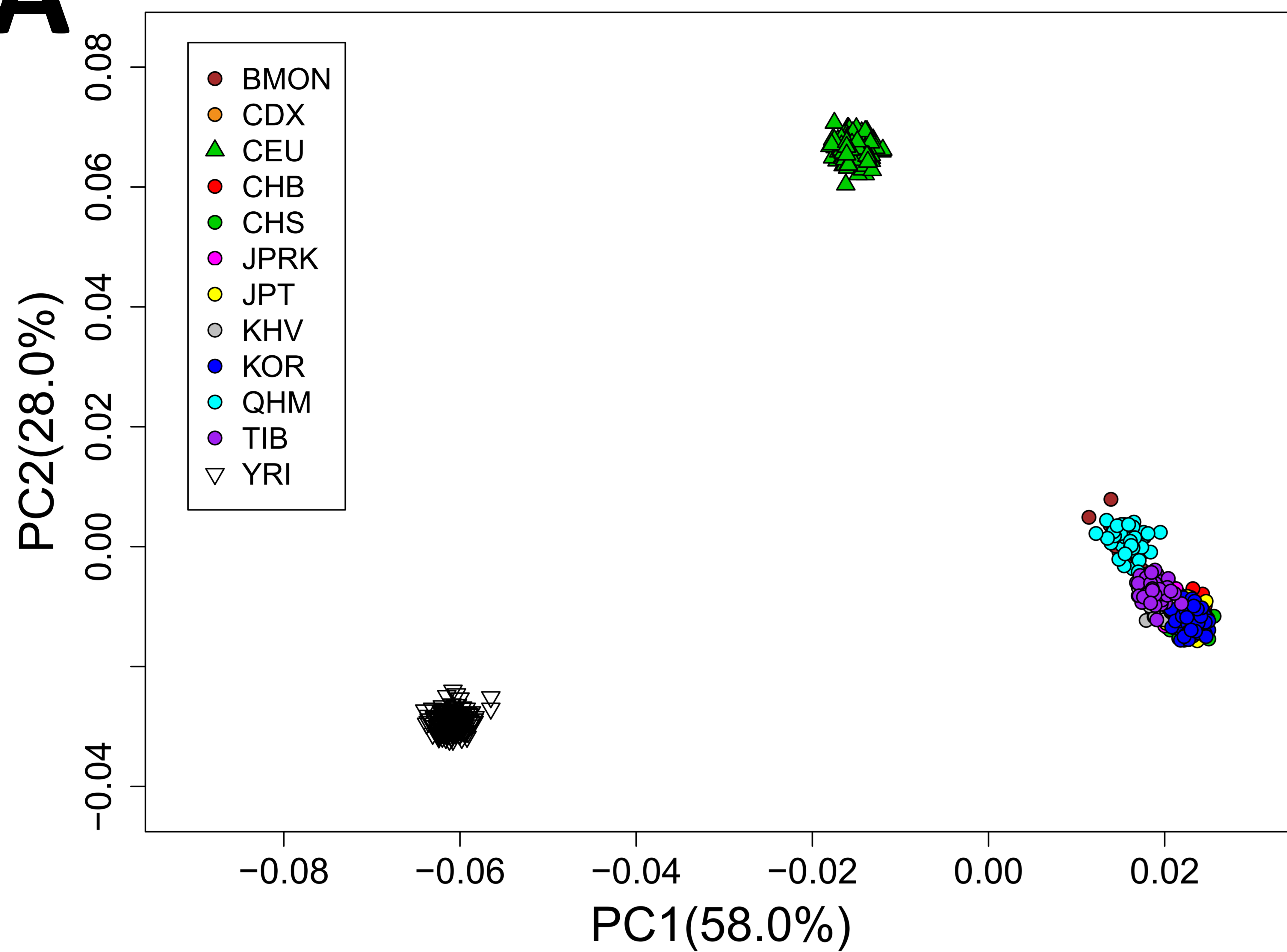**B**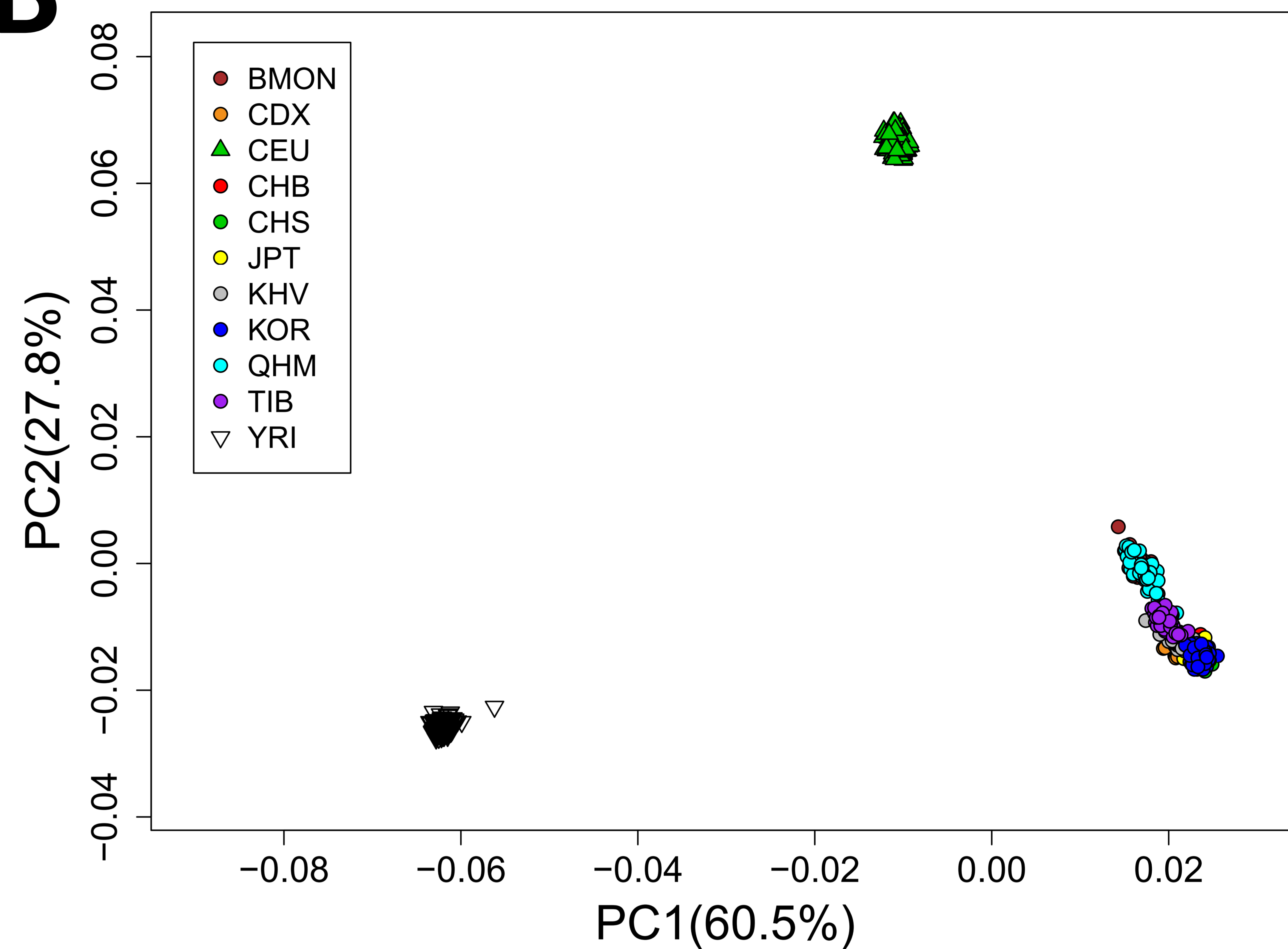**C**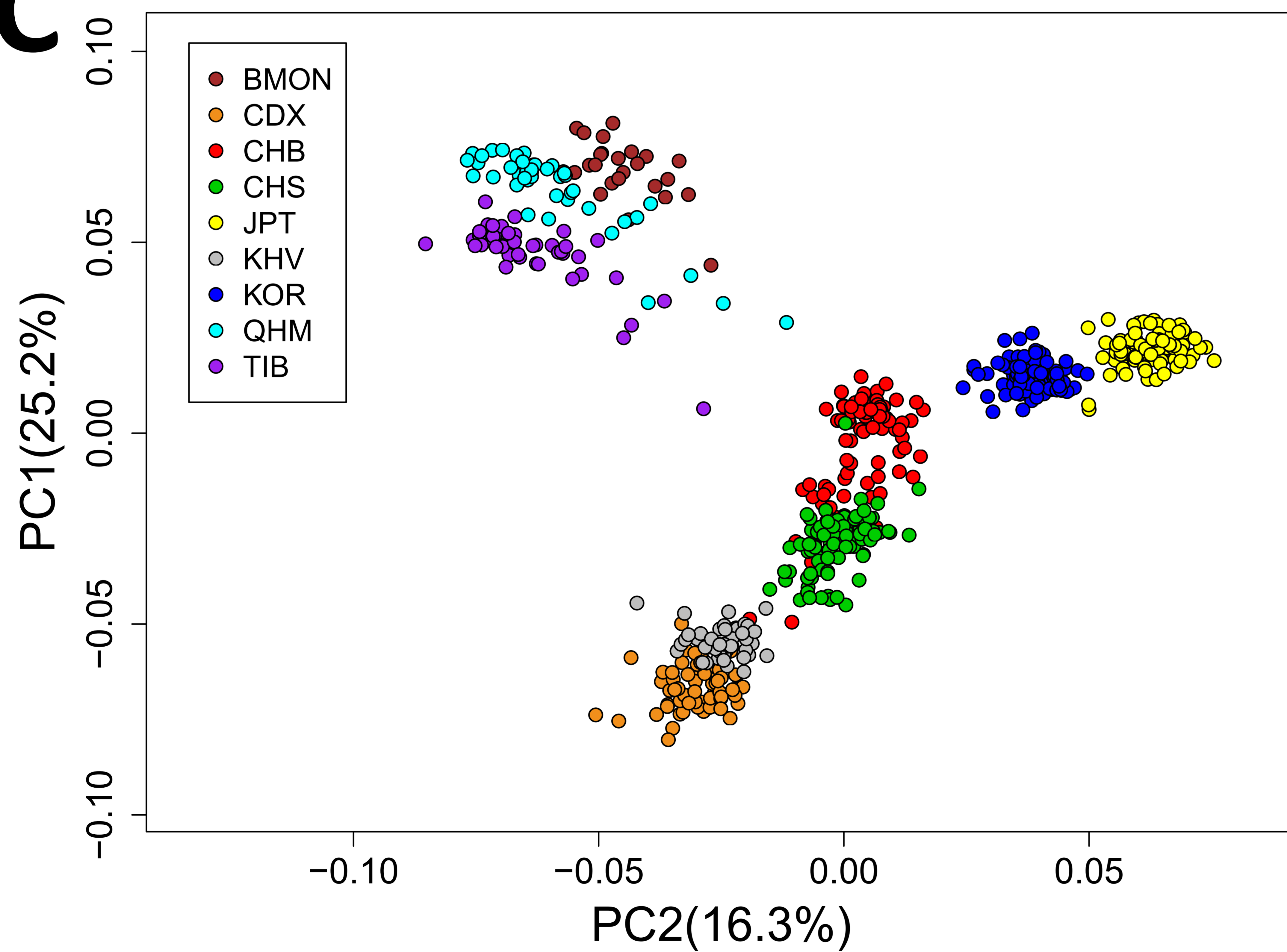**D**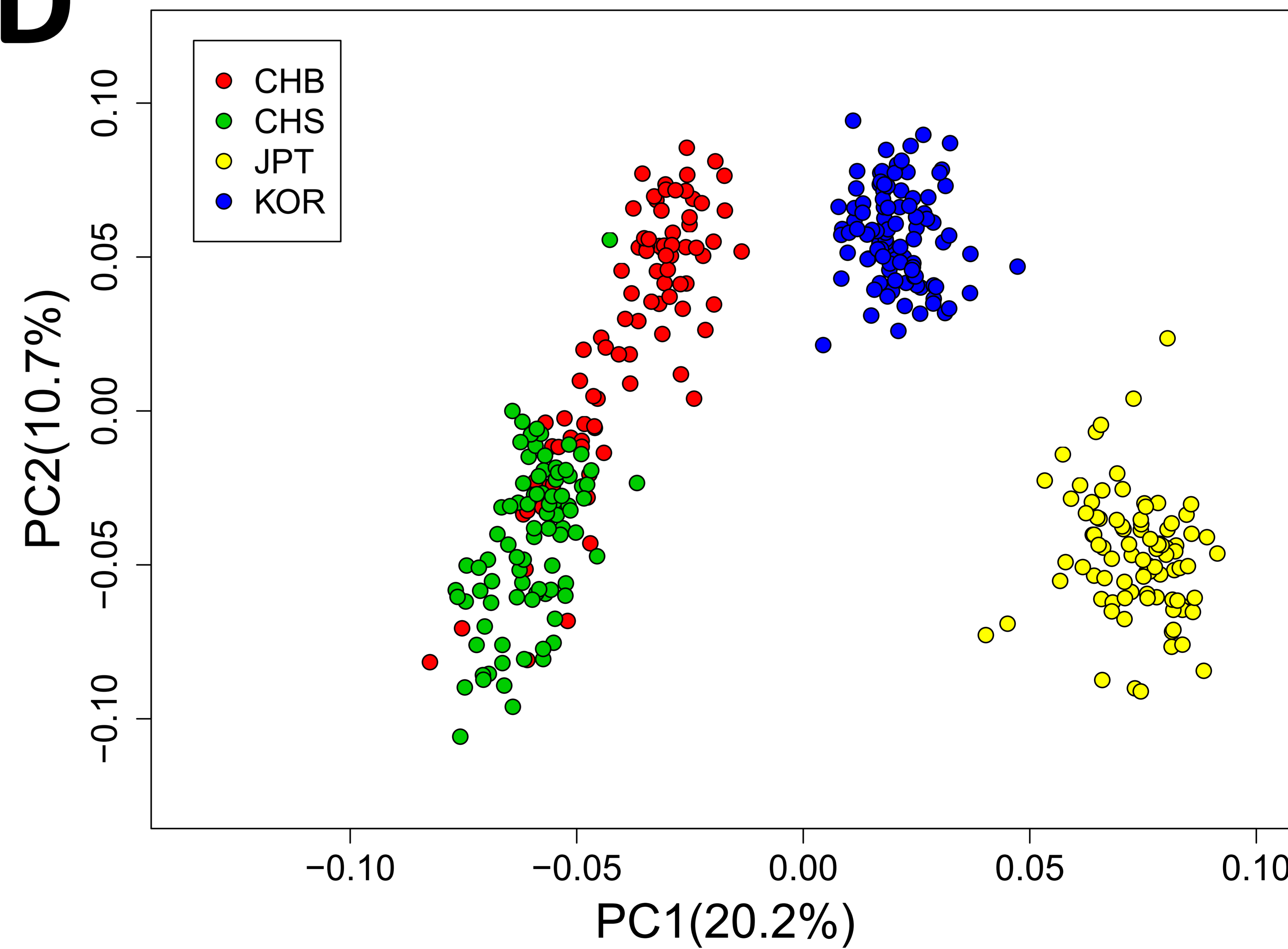

Supplement: Supplementary file 5 — Figure S3. Principle component analysis (PCA). (A~B) PCA results of East Asian groups with CEU and YRI. (C) PCA result of groups within East Asia populations, excluding JPRK individuals for a higher marker density. (D) PCA result of four East Asian populations, including Han Chinese, Japanese and Koreans samples. (PDF 2458 kb) [file 41065_2018_57_MOESM5_ESM.pdf]

**K=3**

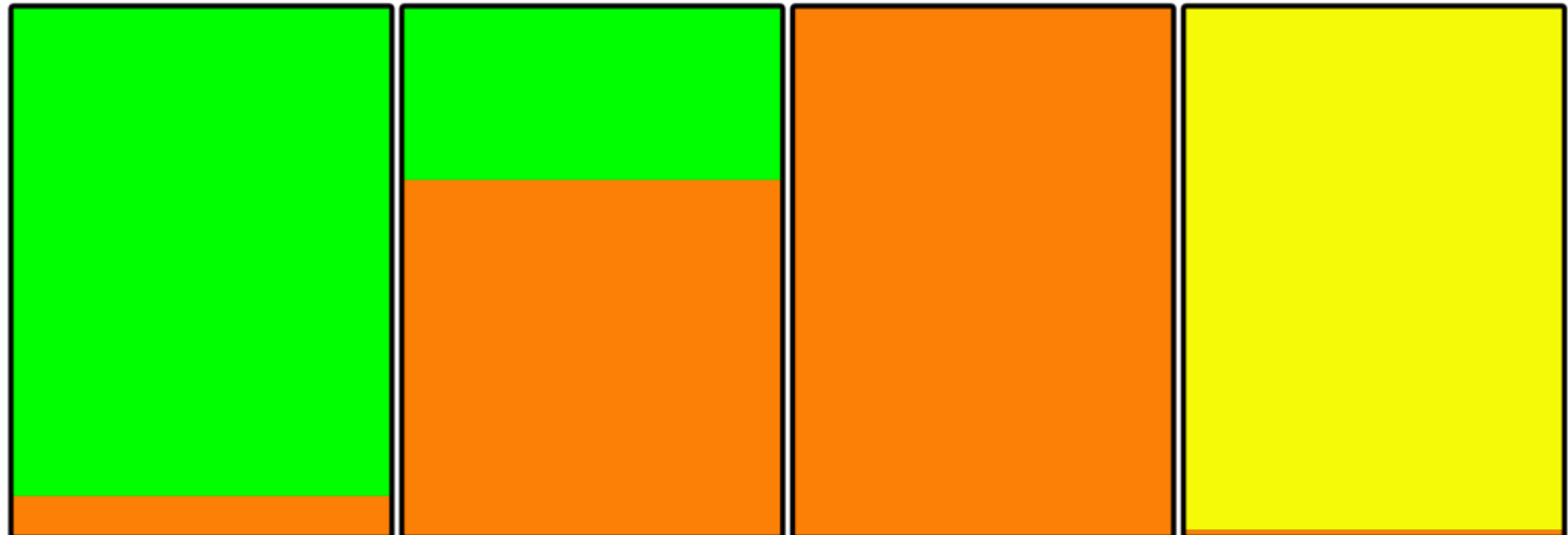

**K=4**

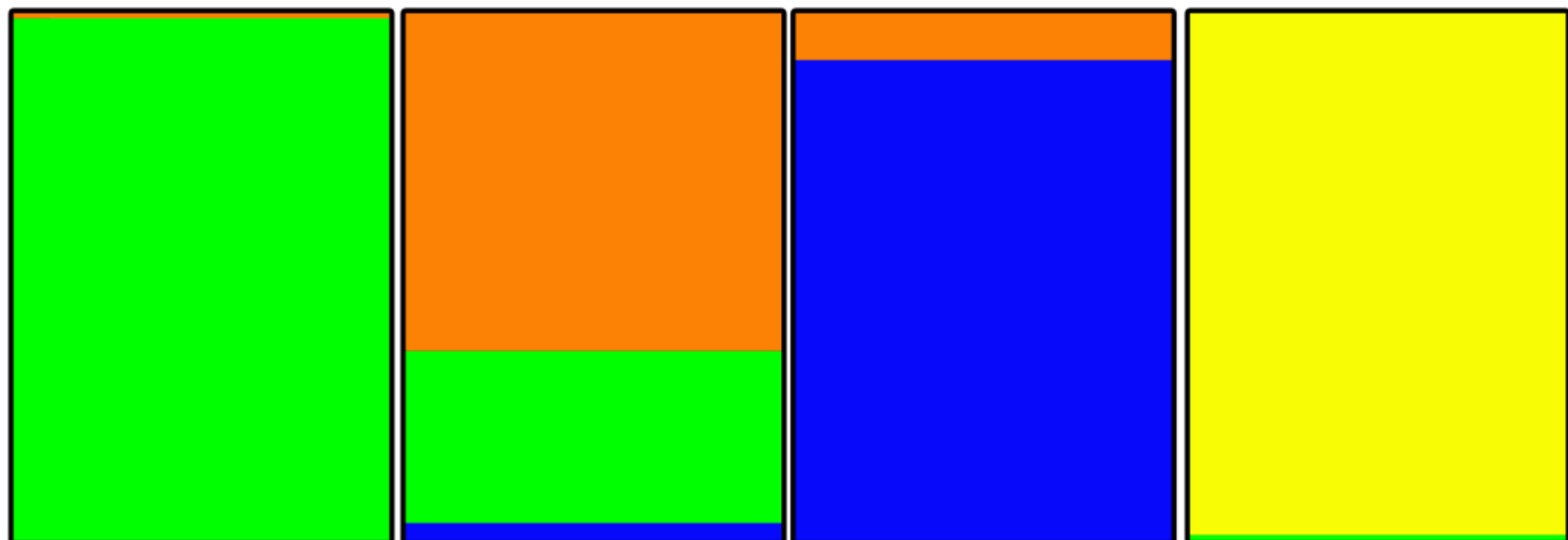

**CHS**

**CHB**

**KOR**

**JPT**

Supplement: Supplementary file 6 — Figure S4. K-means Cluster of PCA results. The results of Figure S3D were used to conduct the K-means cluster. Individuals that cluster together according to the top 10 PCs would be painted by the same color. Results of K = 3 and K = 4 are shown (generated by R 2.15.2). (PDF 116 kb) [file 41065_2018_57_MOESM6_ESM.pdf]

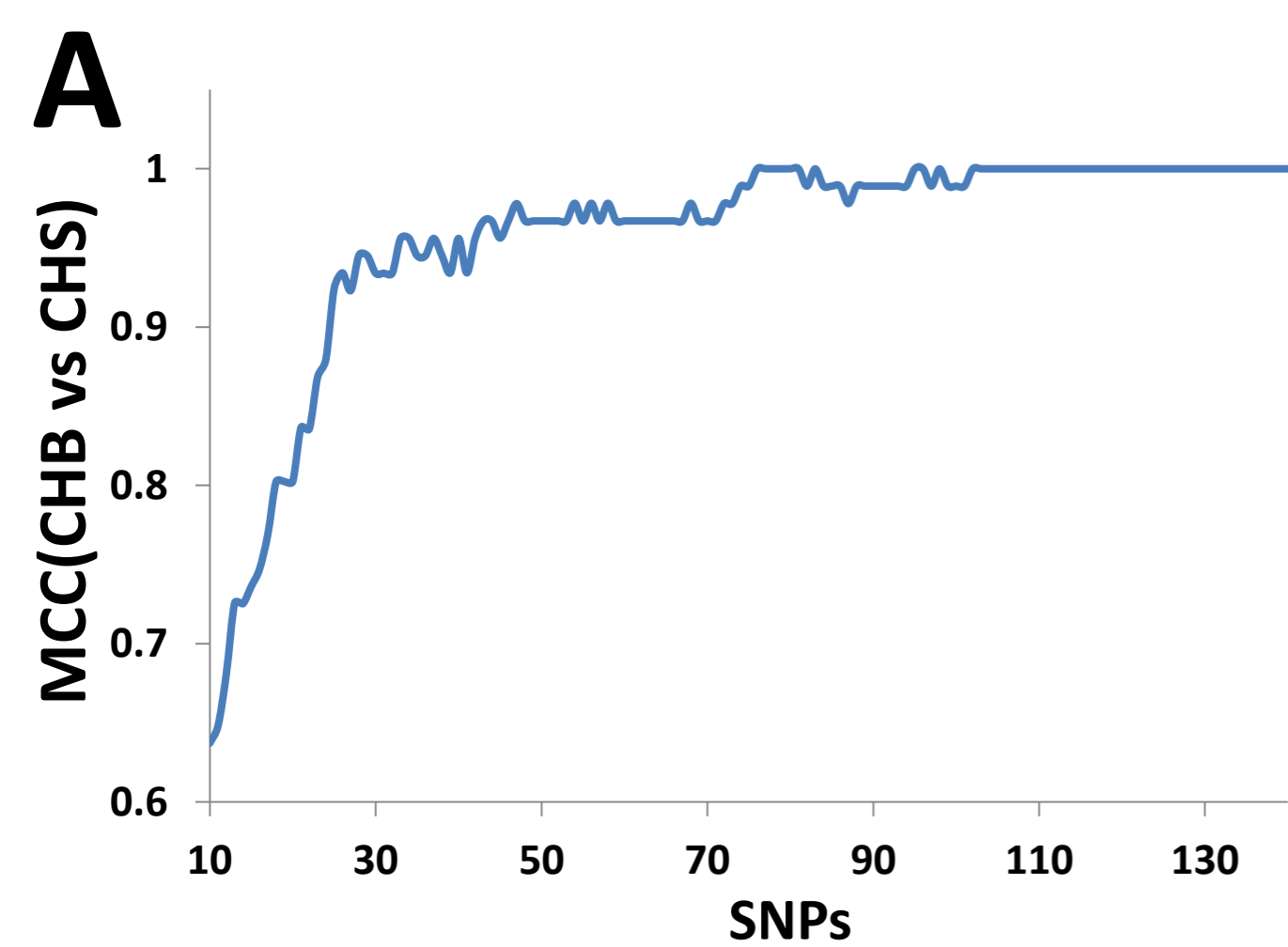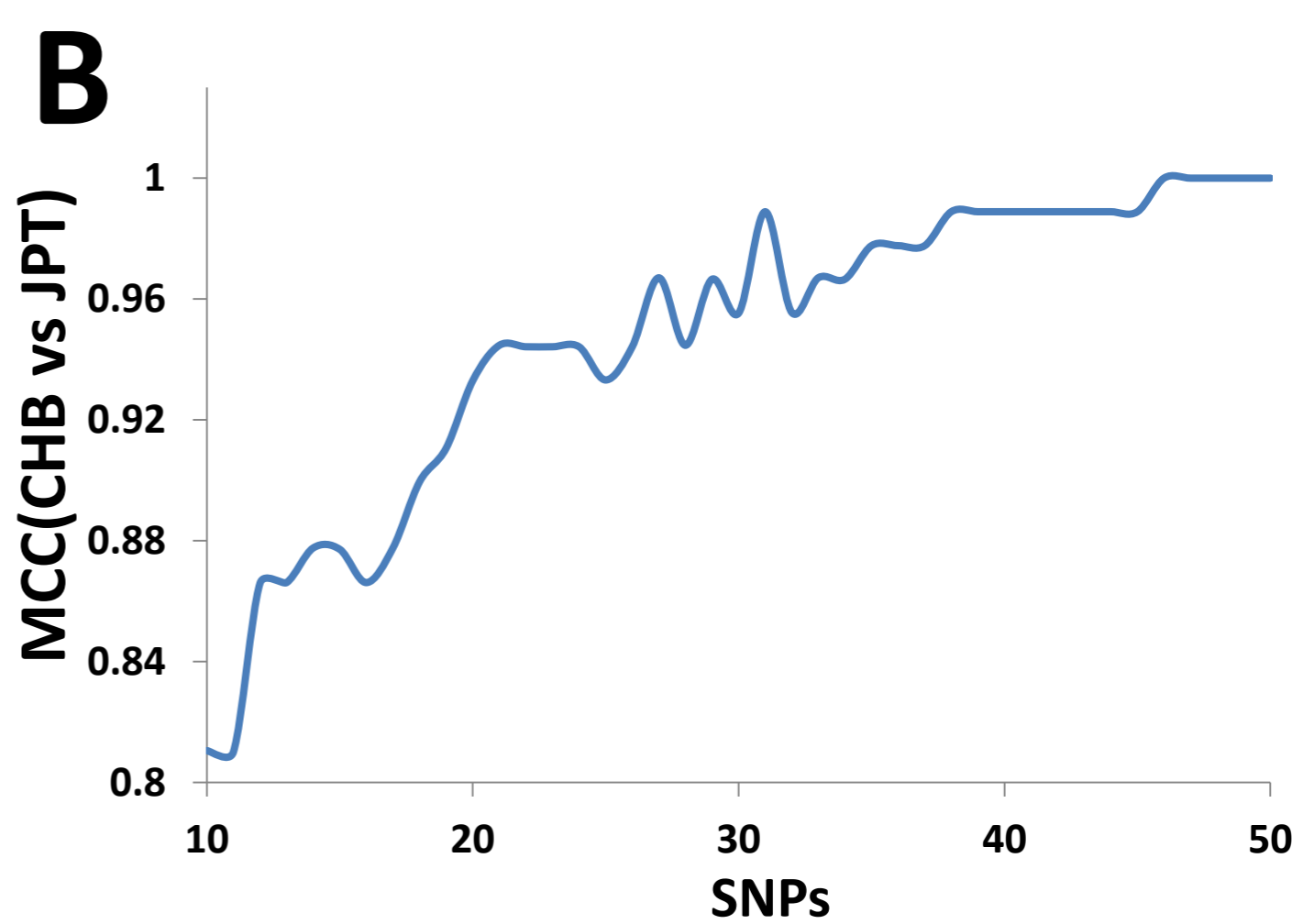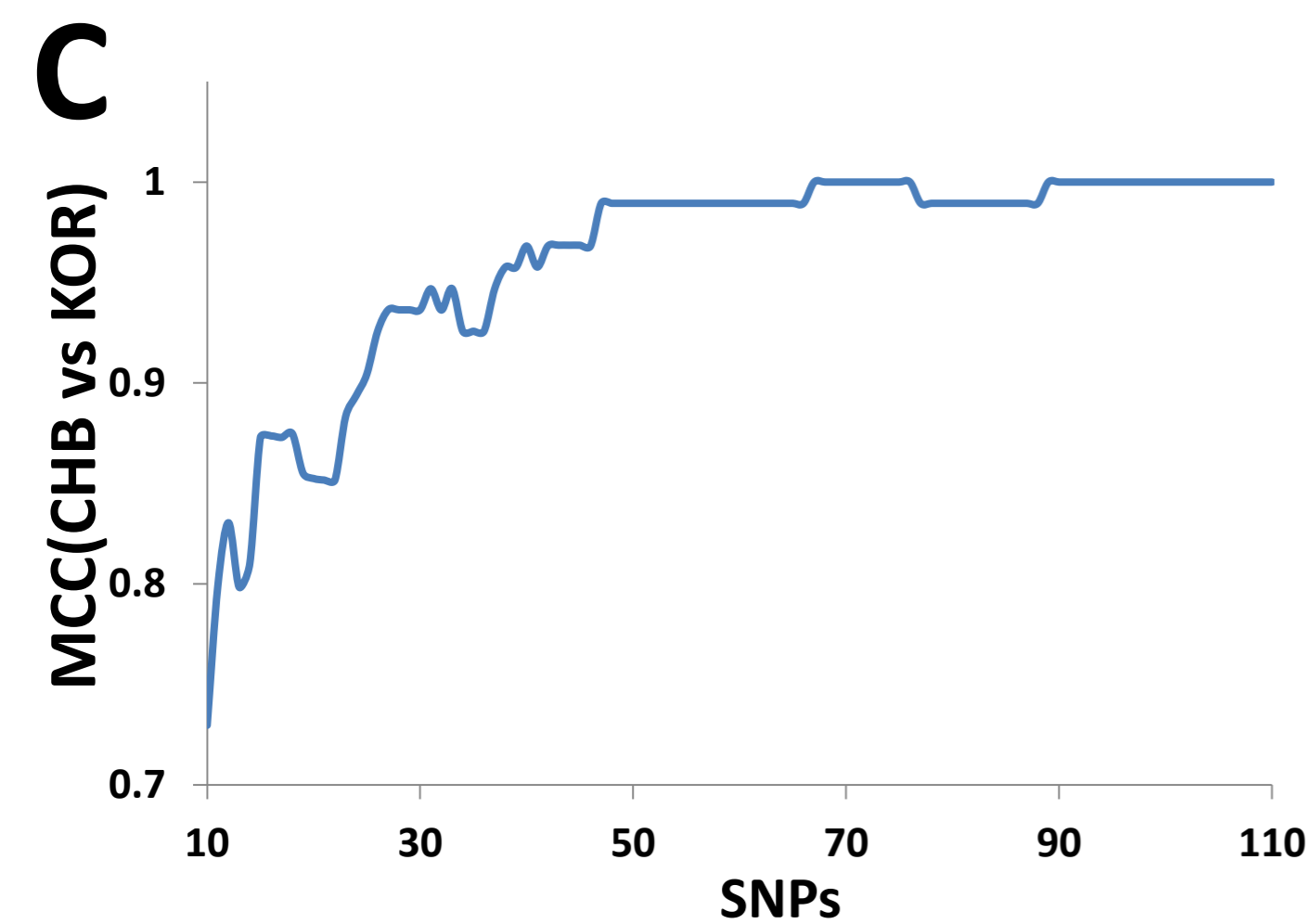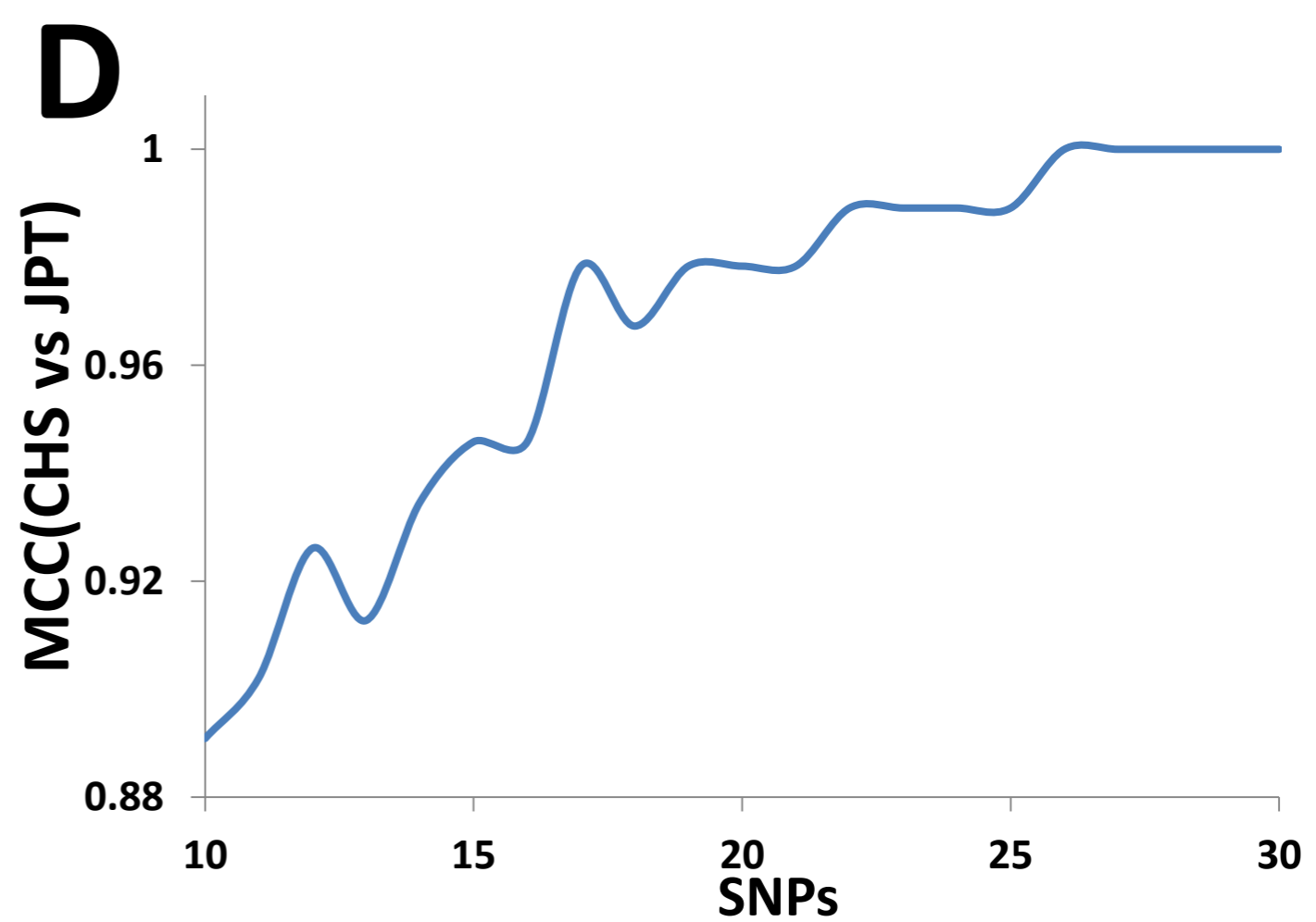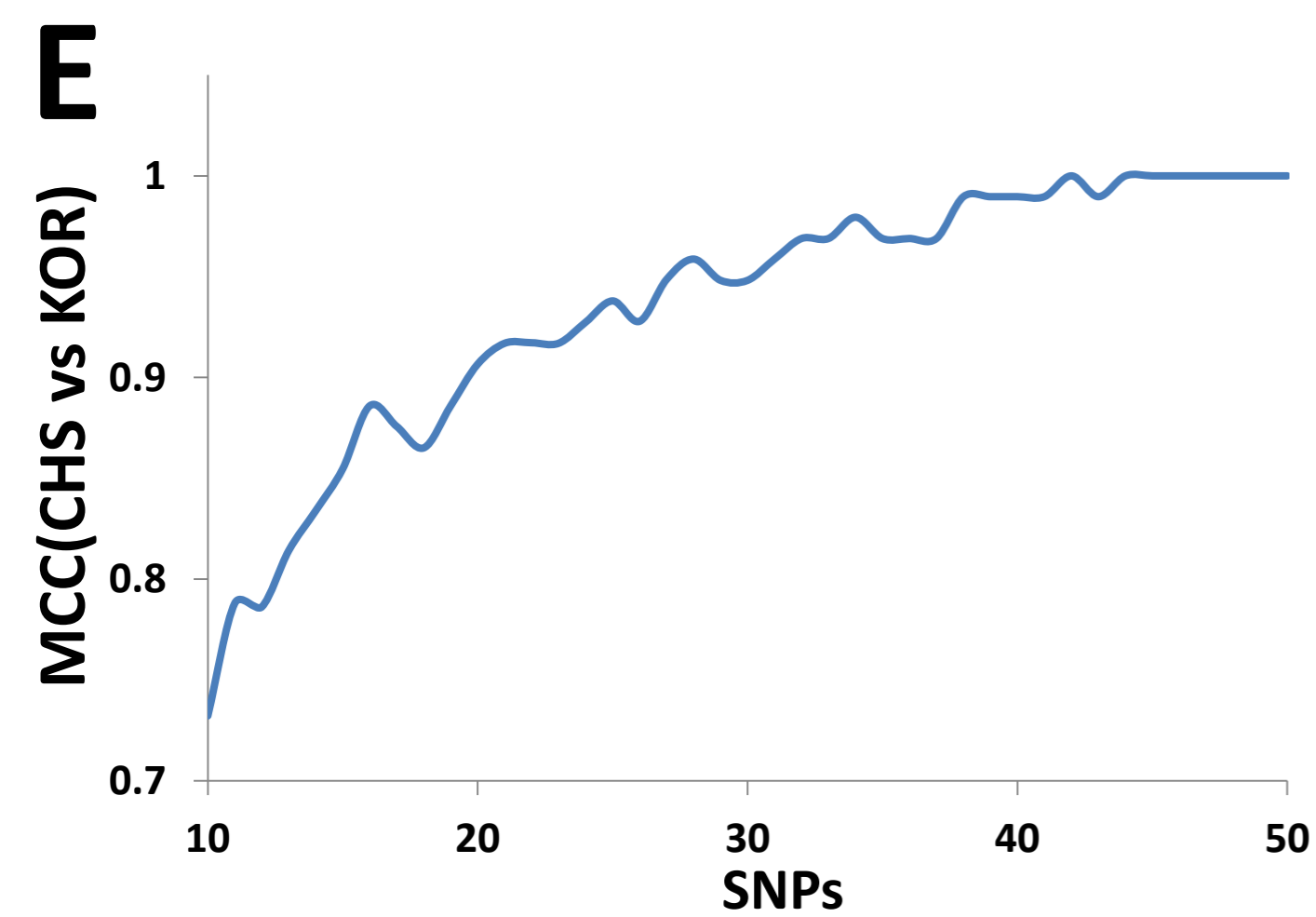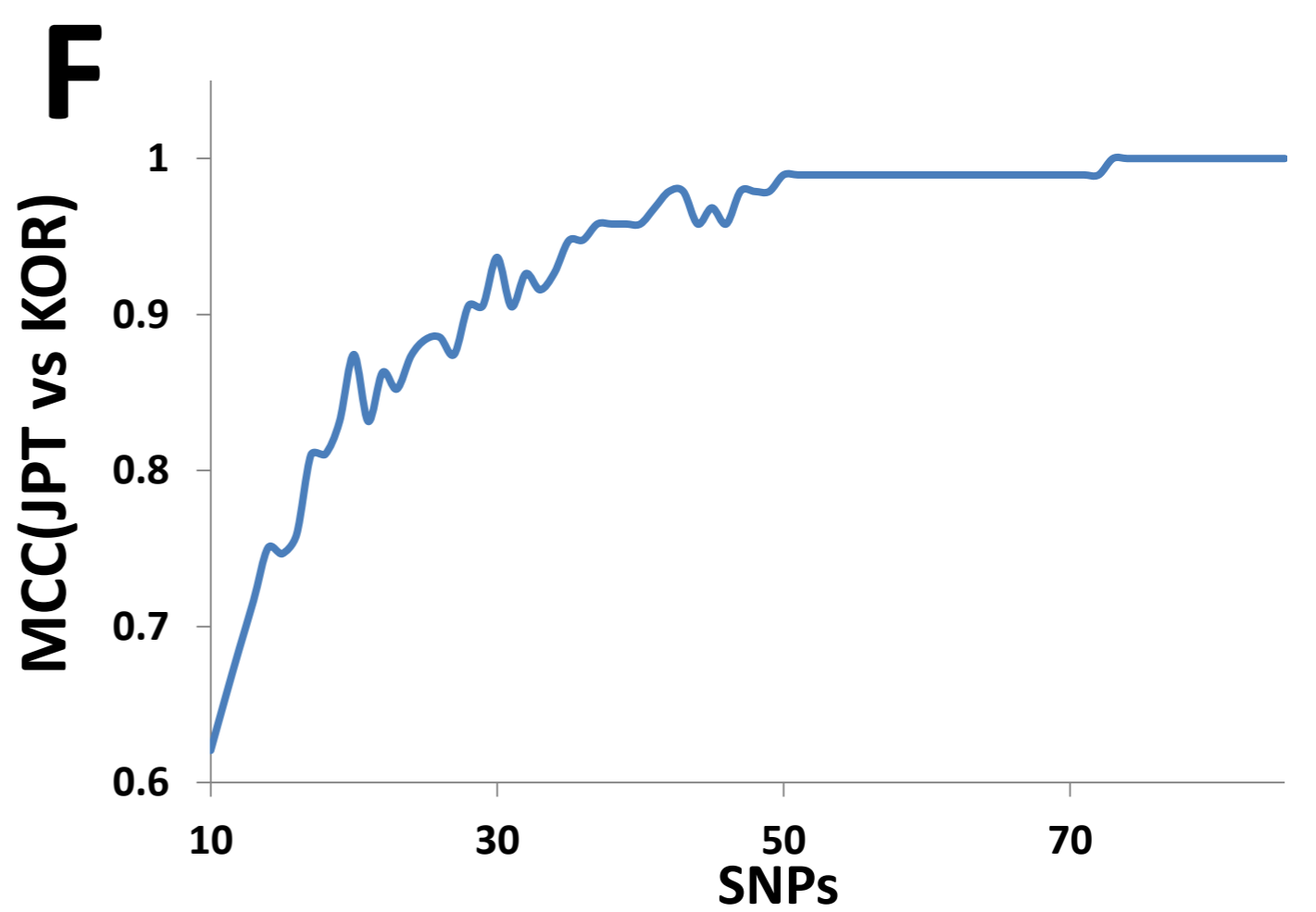

Supplement: Supplementary file 7 — Figure S5. Matthews correlation coefficient (MCC) values increase when more AIMs are used to distinguish individuals. X axis is number of SNPs and Y axis is MCC values. (A) CHB-CHS; (B) CHB-JPT; (C) CHB-KOR; (D) CHS-JPT; (E) CHS-KOR; (F) JPT-KOR (generated by Microsoft Excel 2010). (PDF 99 kb) [file 41065_2018_57_MOESM7_ESM.pdf]

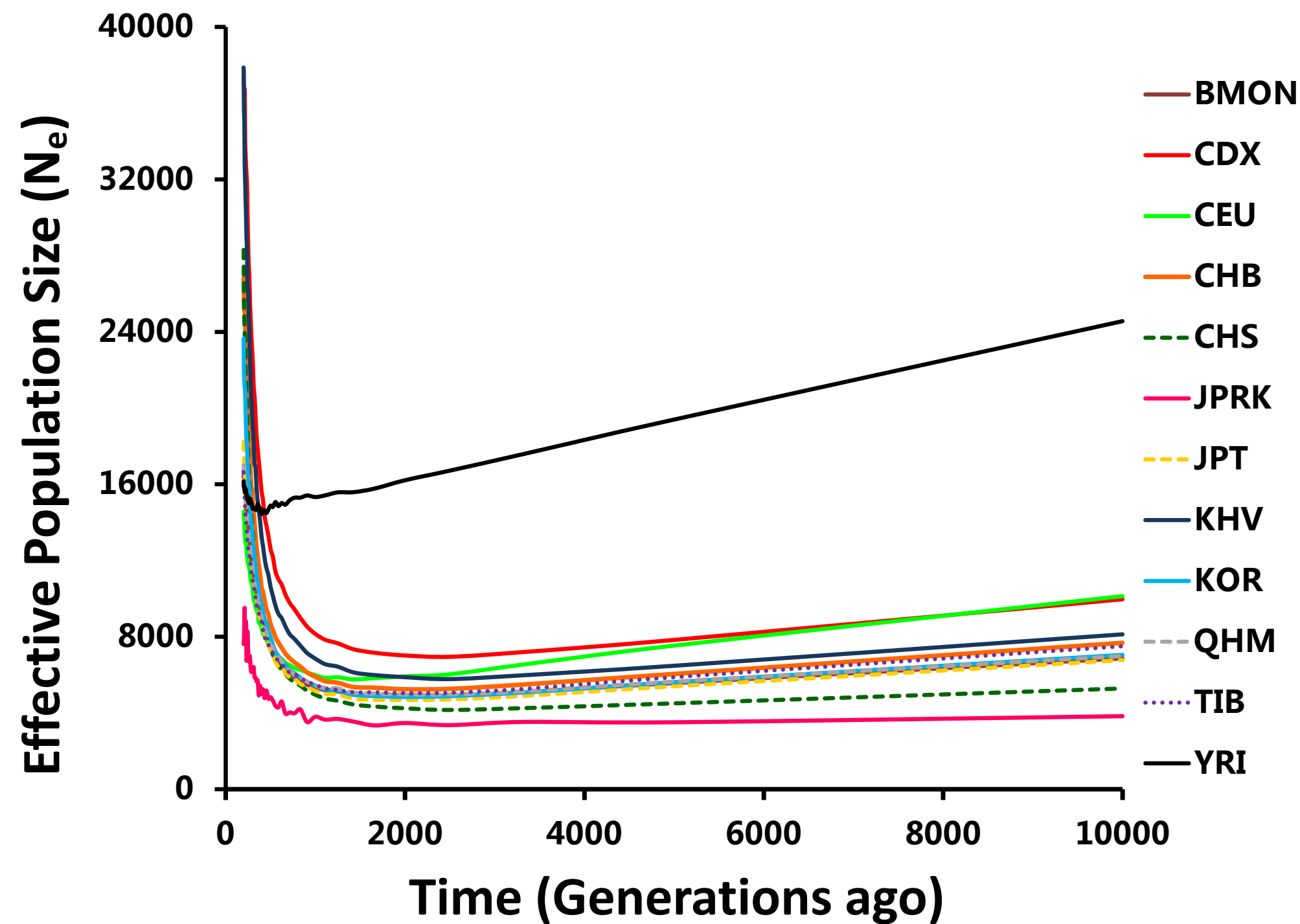

Supplement: Supplementary file 8 — Figure S6. Effective population size varied along time. Results from 10,000 generations ago (GA) to 200 GA are shown (generated by Microsoft Office 2010). (PDF 112 kb) [file 41065_2018_57_MOESM8_ESM.pdf]

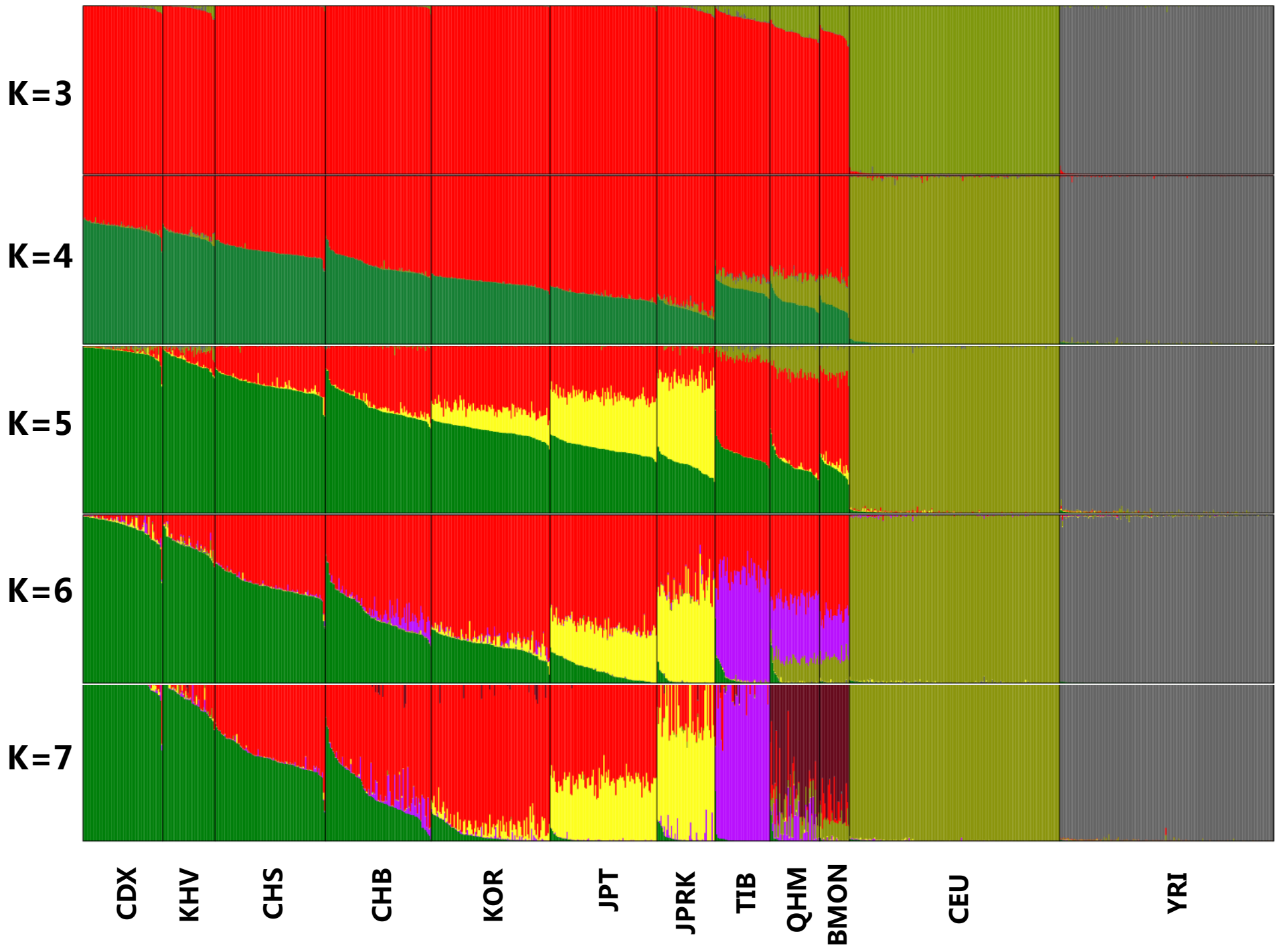

Supplement: Supplementary file 9 — Figure S7. STRUCTURE analysis of East Asian samples with worldwide populations. Results from K = 3 to K = 7 are shown. Each vertical bar represents an individual and each color stands for a genetic component (generated by R 2.15.2). (PDF 141 kb) [file 41065_2018_57_MOESM9_ESM.pdf]

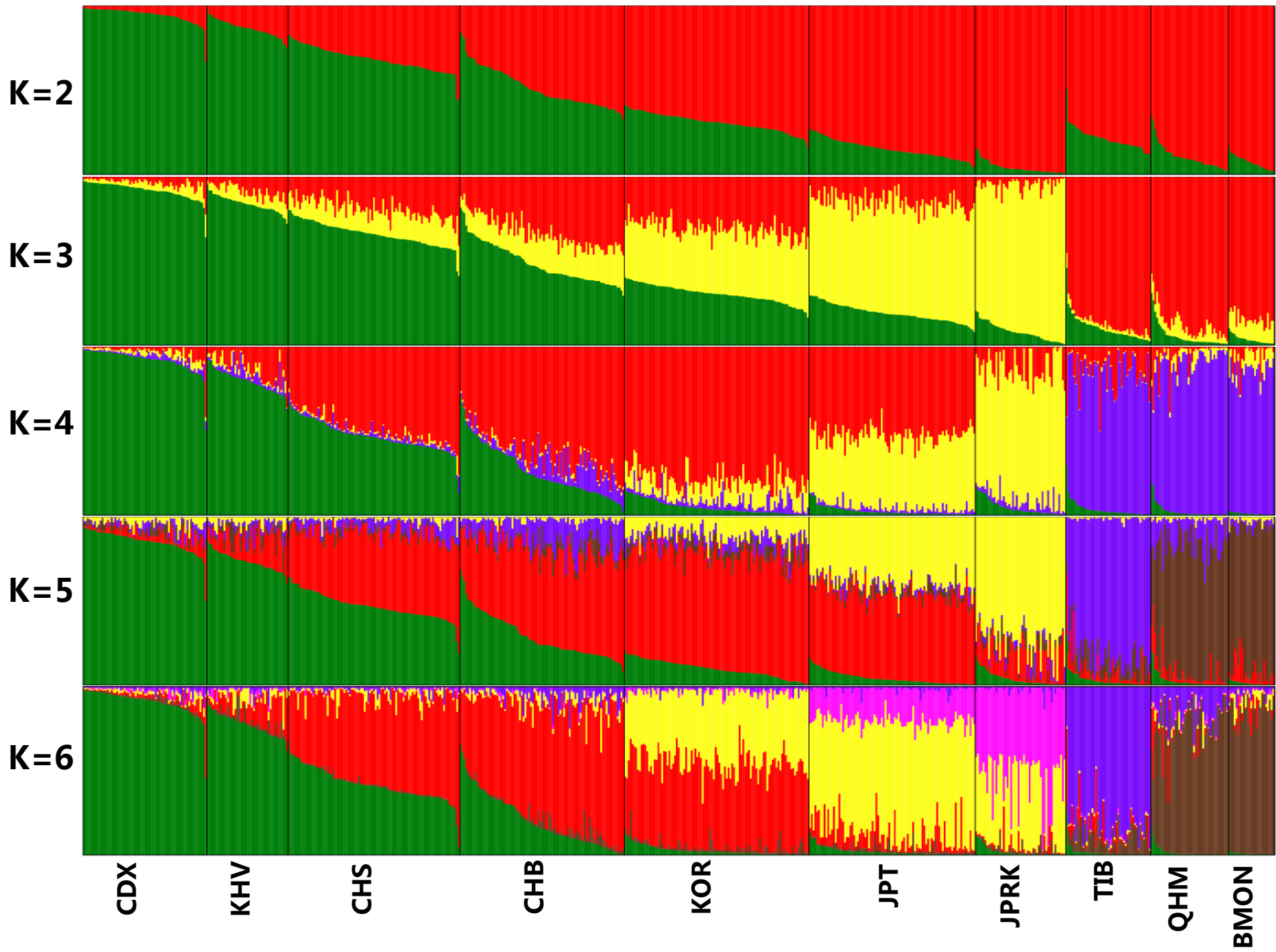

Supplement: Supplementary file 10 — Figure S8. STRUCTURE Analysis of ten East Asian populations. Results from K = 2 to K = 6 are shown. Each vertical bar represents an individual and each color stands for a genetic component (generated by R 2.15.2). (PDF 152 kb) [file 41065_2018_57_MOESM10_ESM.pdf]

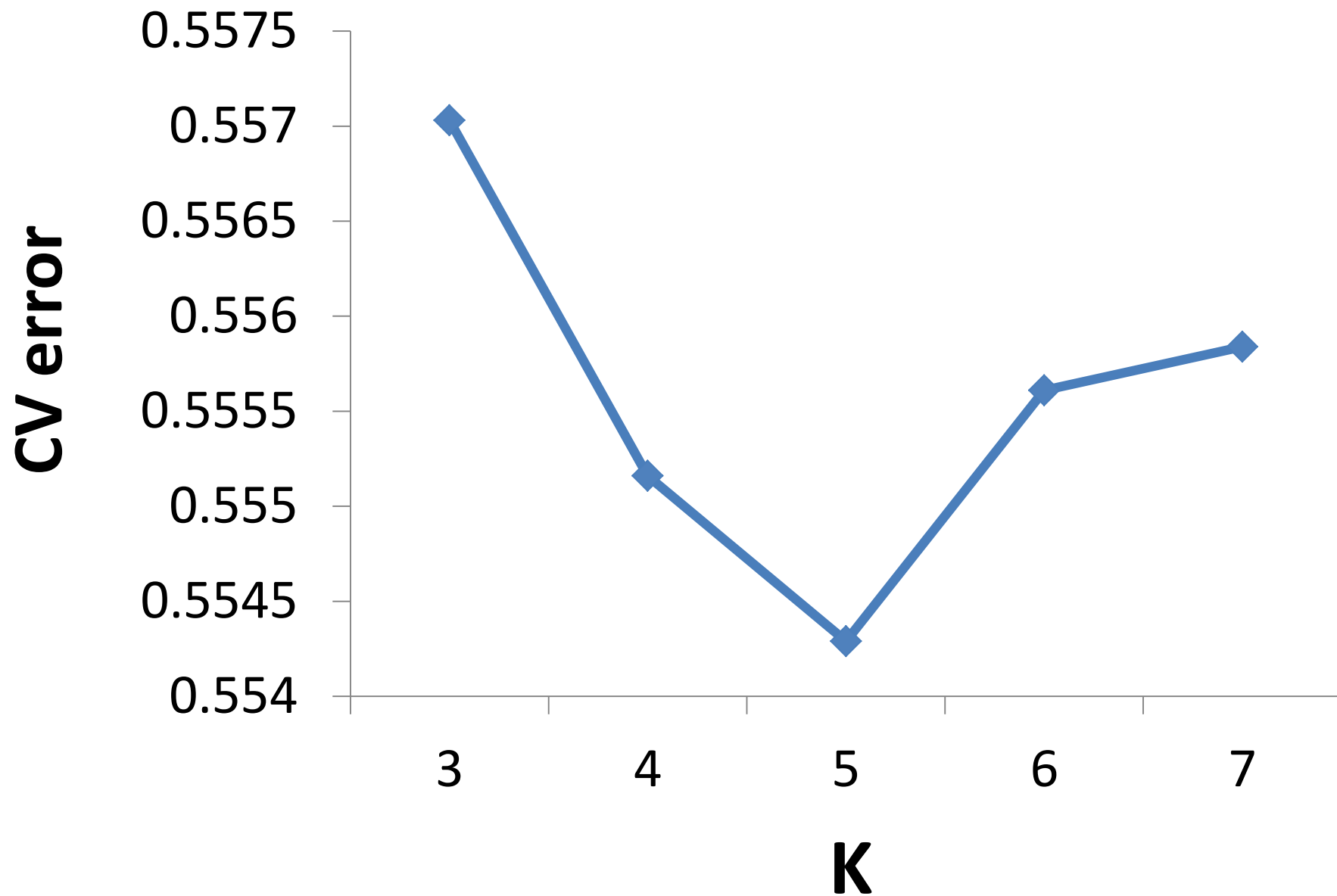

Supplement: Supplementary file 11 — Figure S9. Cross-validation (CV) plot for the Admixture analysis. Result of K = 3 to K = 7 are shown. (PDF 158 kb) [file 41065_2018_57_MOESM11_ESM.pdf]

A

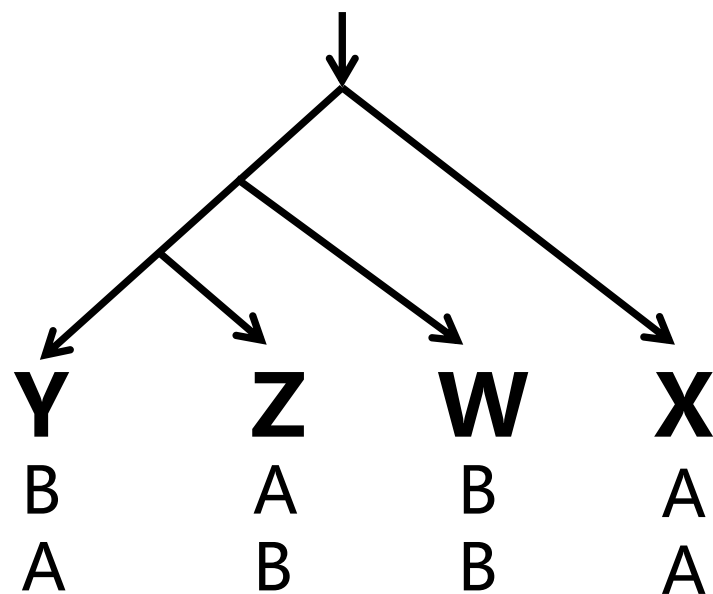

B

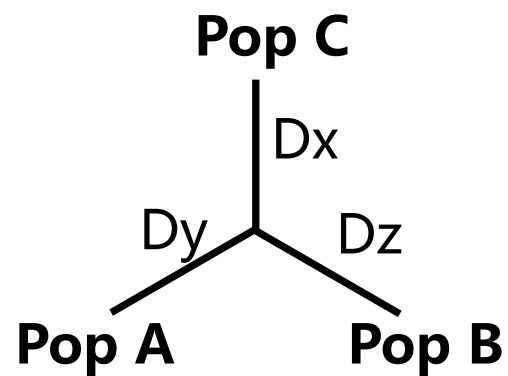

C

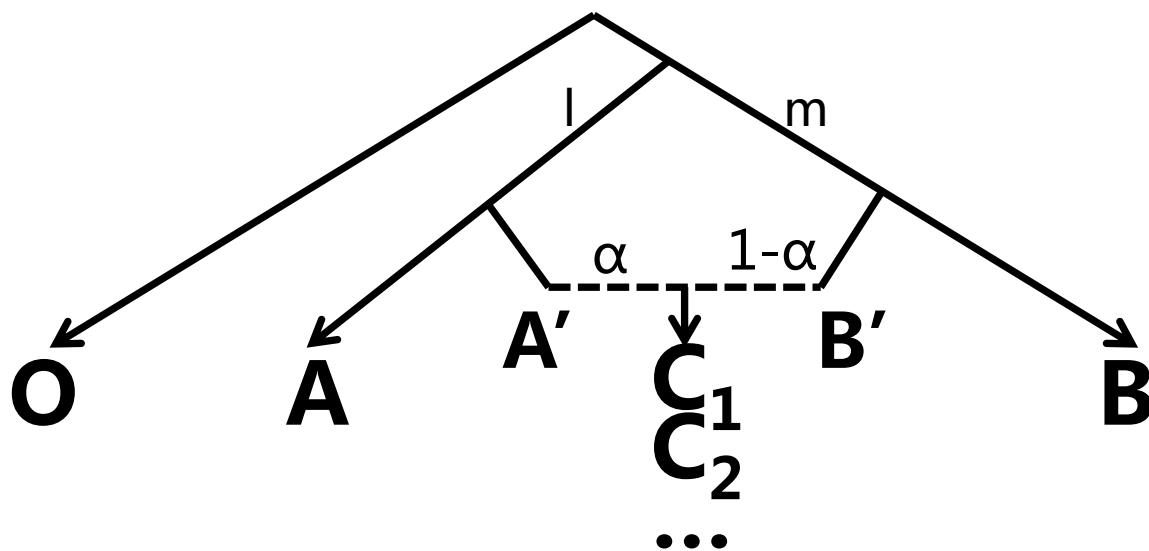

Supplement: Supplementary file 13 — Figure S11. Models used in gene flow study. (A) Model of D test. X is an out group, we put CEU or YRI in X; W is the contributor while Y and Z are the receivers. By comparing the two allelic patterns BABA and ABBA, we can infer the relationship of scale of gene flow between W to Y and W to Z. (PDF 30 kb) [file 41065_2018_57_MOESM13_ESM.pdf]

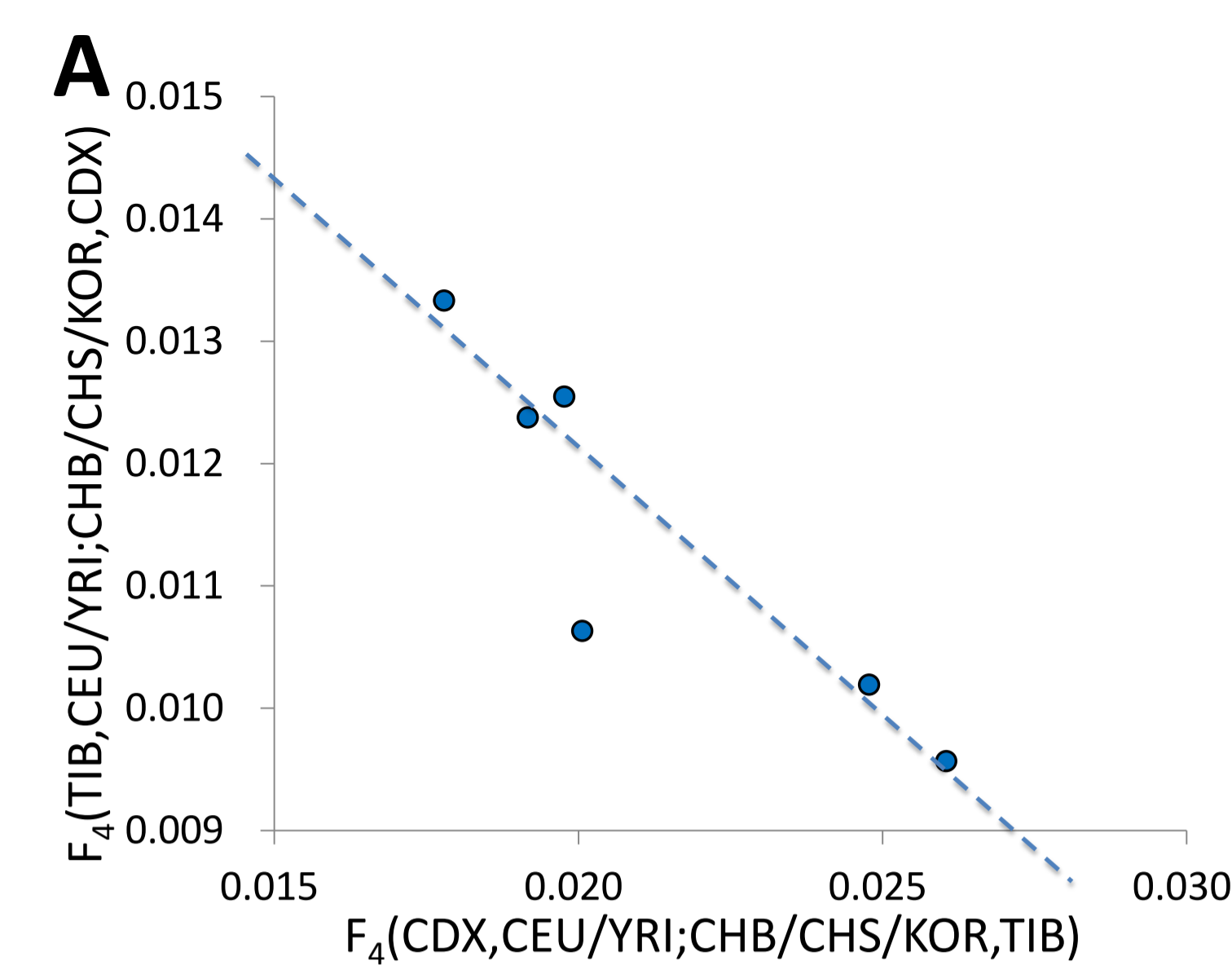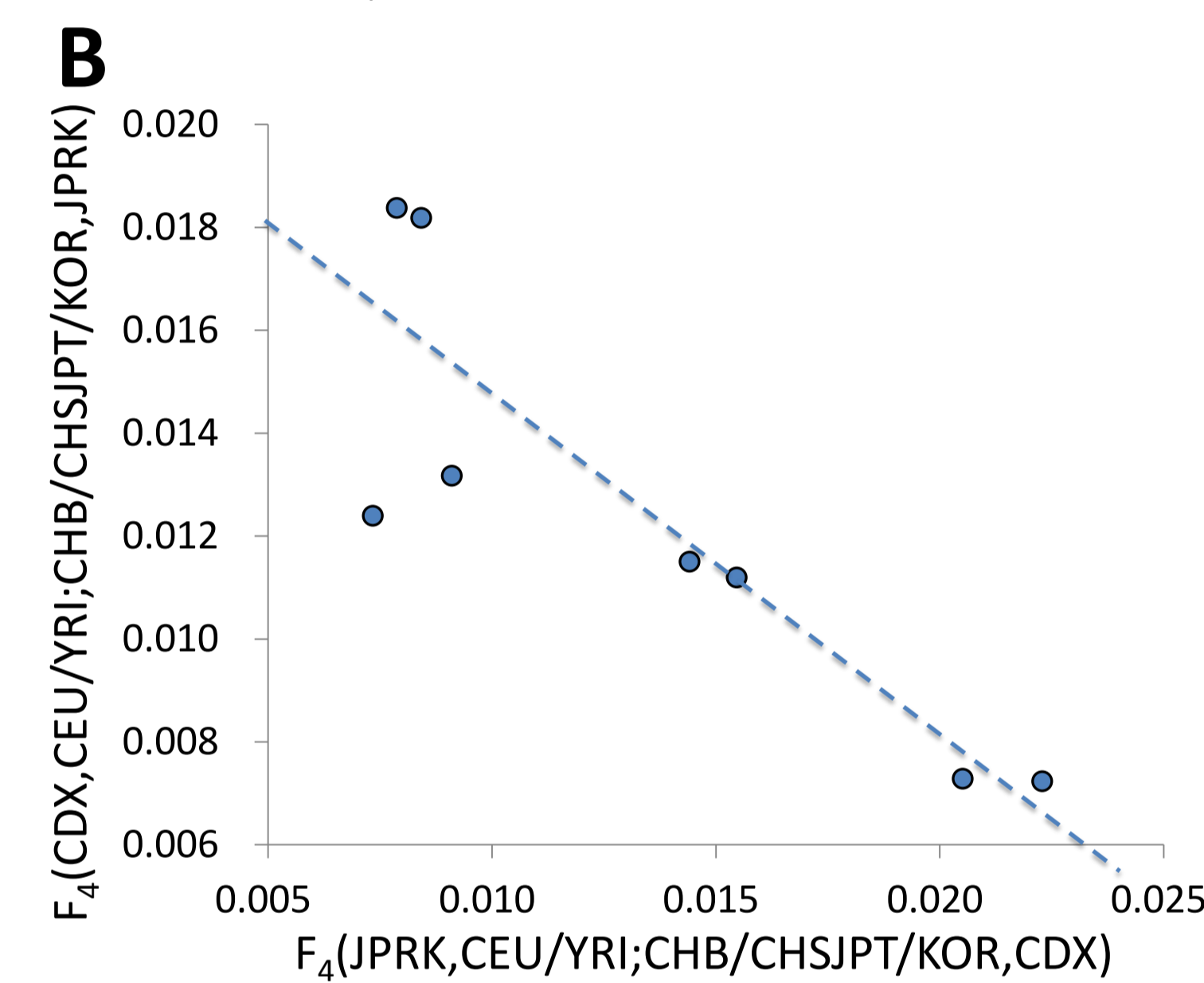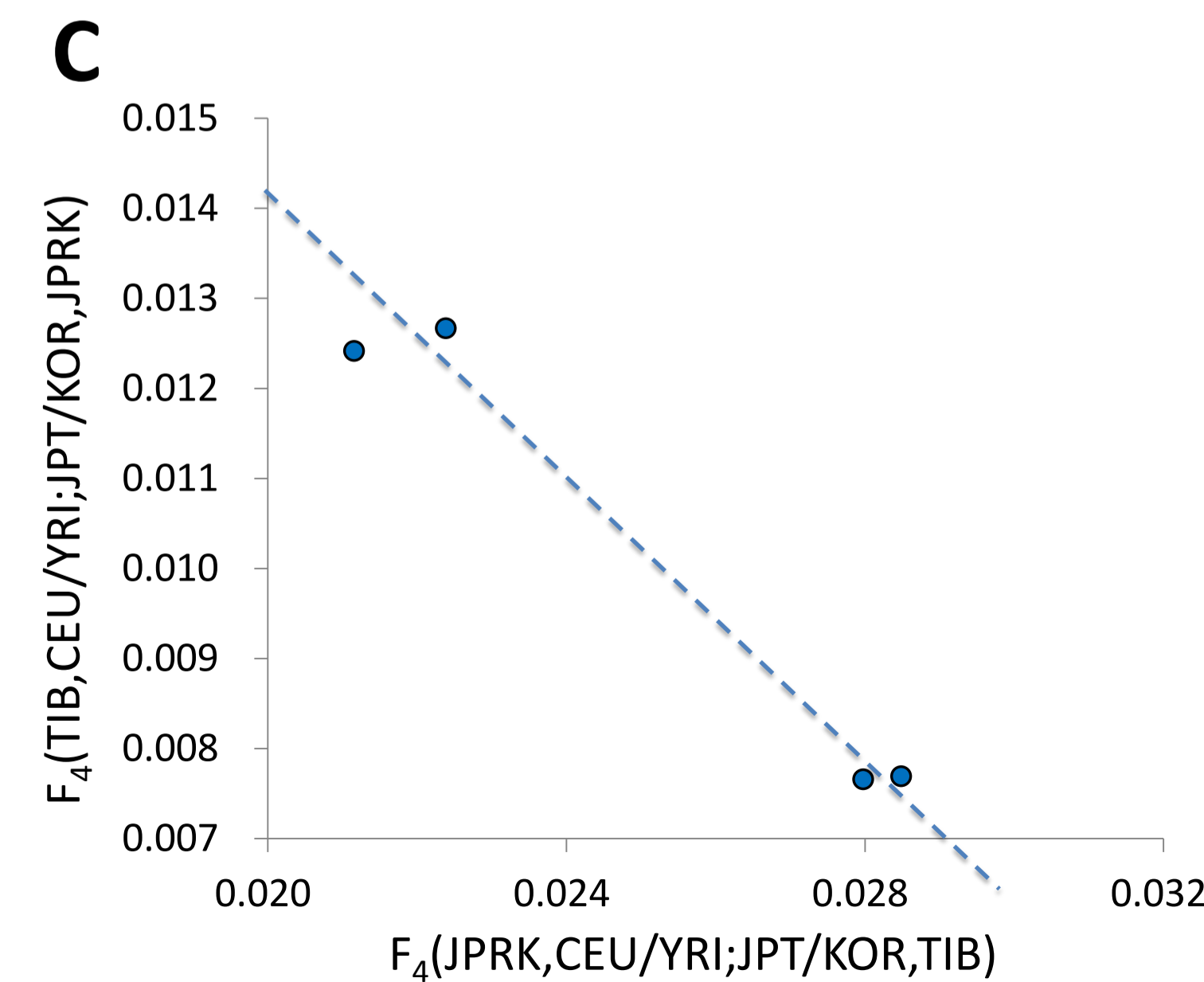

Supplement: Supplementary file 14 — Figure S10. Results of linear regression in F4 test. For each group (Outgroup O, donate population A, donate population B, target population X), X axis is F4(B, O; X, A), Y axis is F4(A, O; X, B). (A) Outgroup is YRI or CEU, donate populations are TIB and CDX, target population is Han Chinese or KOR; (B) Outgroup is YRI or CEU, donate populations are CDX and JPRK, target population is Han Chinese or JPT or KOR; (C) Outgroup is YRI or CEU, donate populations are TIB and JPRK, target population is JPT or KOR (generated by Microsoft Excel 2010). (PDF 226 kb) [file 41065_2018_57_MOESM14_ESM.pdf]
