# Supplementary material for: Genetic structure, divergence and admixture of Han Chinese, Japanese and Korean populations
Source: Hereditas. 2018 Apr 6;155:19. doi: 10.1186/s41065-018-0057-5 (PMC5889524; doi:10.1186/s41065-018-0057-5)
Supplement: Supplementary file 2 — Table S2. Global FST values between East Asian populations and world-wide groups. (DOCX 16 kb) [file 41065_2018_57_MOESM2_ESM.docx]

**Table S2 | Global F_ST_ values between East Asian populations and world-wide groups.**

| **F_ST_** | **BMON** | **CDX** | **CEU** | **CHB** | **CHS** | **JPRK** | **JPT** | **KHV** | **KOR** | **QHM** | **TIB** | **YRI** |
| --- | --- | --- | --- | --- | --- | --- | --- | --- | --- | --- | --- | --- |
| **BMON** | 0 | 0.0273 (0.0271-0.0275) | 0.0838 (0.0835-0.0842) | 0.0152 (0.0151-0.0153) | 0.0190 (0.0189-0.0191) | 0.0241 (0.0232-0.0250) | 0.0162 (0.0161-0.0163) | 0.0239 (0.0237-0.0241) | 0.0139 (0.0138-0.0140) | 0.0053 (0.0052-0.0054) | 0.0172 (0.0171-0.0174) | 0.1605 (0.1599-0.1611) |
| **CDX** | 0.0273 (0.0271-0.0275) | 0 | 0.1093 (0.1088-0.1097) | 0.0093 (0.0093-0.0094) | 0.0054 (0.0053-0.0054) | 0.0264 (0.0258-0.0269) | 0.0175 (0.0174-0.0176) | 0.0024 (0.0024-0.0025) | 0.0143 (0.0142-0.0144) | 0.0232 (0.0230-0.0234) | 0.0238 (0.0237-0.0240) | 0.1725 (0.1718-0.1730) |
| **CEU** | 0.0838 (0.0835-0.0842) | 0.1093 (0.1088-0.1097) | 0 | 0.1088 (0.1085-0.1092) | 0.1089 (0.1086-0.1093) | 0.1109 (0.1083-0.1133) | 0.1098 (0.1094-0.1101) | 0.1059 (0.1055-0.1063) | 0.1109 (0.1106-0.1113) | 0.0830 (0.0827-0.0833) | 0.1031 (0.1027-0.1035) | 0.1522 (0.1517-0.1526) |
| **CHB** | 0.0152 (0.0151-0.0153) | 0.0093 (0.0093-0.0094) | 0.1088 (0.1085-0.1092) | 0 | 0.0014 (0.0014-0.0014) | 0.0165 (0.0159-0.0170) | 0.0070 (0.0069-0.0070) | 0.0071 (0.0070-0.0071) | 0.0026 (0.0025-0.0026) | 0.0115 (0.0114-0.0116) | 0.0114 (0.0113-0.0114) | 0.1747 (0.1741-0.1752) |
| **CHS** | 0.0190 (0.0189-0.0191) | 0.0054 (0.0053-0.0054) | 0.1089 (0.1086-0.1093) | 0.0014 (0.0014-0.0014) | 0 | 0.0183 (0.0177-0.0189) | 0.0090 (0.0089-0.0090) | 0.0037 (0.0037-0.0038) | 0.0048 (0.0047-0.0048) | 0.0150 (0.0149-0.0151) | 0.0148 (0.0147-0.0149) | 0.1731 (0.1725-0.1737) |
| **JPRK** | 0.0241 (0.0232-0.0250) | 0.0264 (0.0258-0.0269) | 0.1109 (0.1083-0.1133) | 0.0165 (0.0159-0.0170) | 0.0183 (0.0177-0.0189) | 0 | 0.0062 (0.0059-0.0065) | 0.0235 (0.0230-0.0239) | 0.0133 (0.0128-0.0138) | 0.0215 (0.0208-0.0223) | 0.0258 (0.0250-0.0265) | 0.1738 (0.1706-0.1771) |
| **JPT** | 0.0162 (0.0161-0.0163) | 0.0175 (0.0174-0.0176) | 0.1098 (0.1094-0.1101) | 0.0070 (0.0069-0.0070) | 0.0090 (0.0089-0.0090) | 0.0062 (0.0059-0.0065) | 0 | 0.0148 (0.0147-0.0149) | 0.0033 (0.0032-0.0033) | 0.0143 (0.0142-0.0144) | 0.0177 (0.0176-0.0178) | 0.1756 (0.1751-0.1762) |
| **KHV** | 0.0239 (0.0237-0.0241) | 0.0024 (0.0024-0.0025) | 0.1059 (0.1055-0.1063) | 0.0071 (0.0070-0.0071) | 0.0037 (0.0037-0.0038) | 0.0235 (0.0230-0.0239) | 0.0148 (0.0147-0.0149) | 0 | 0.0117 (0.0116-0.0118) | 0.0200 (0.0198-0.0201) | 0.0212 (0.0211-0.0213) | 0.1715 (0.1709-0.1720) |
| **KOR** | 0.0139 (0.0138-0.0140) | 0.0143 (0.0142-0.0144) | 0.1109 (0.1106-0.1113) | 0.0026 (0.0025-0.0026) | 0.0048 (0.0047-0.0048) | 0.0133 (0.0128-0.0138) | 0.0033 (0.0032-0.0033) | 0.0117 (0.0116-0.0118) | 0 | 0.0115 (0.0114-0.0116) | 0.0136 (0.0135-0.0136) | 0.1757 (0.1752-0.1763) |
| **QHM** | 0.0053 (0.0052-0.0054) | 0.0232 (0.0230-0.0234) | 0.0830 (0.0827-0.0833) | 0.0115 (0.0114-0.0116) | 0.0150 (0.0149-0.0151) | 0.0215 (0.0208-0.0223) | 0.0143 (0.0142-0.0144) | 0.0200 (0.0198-0.0201) | 0.0115 (0.0114-0.0116) | 0 | 0.0116 (0.0115-0.0117) | 0.1598 (0.1593-0.1604) |
| **TIB** | 0.0172 (0.0171-0.0174) | 0.0238 (0.0237-0.0240) | 0.1031 (0.1027-0.1035) | 0.0114 (0.0113-0.0114) | 0.0148 (0.0147-0.0149) | 0.0258 (0.0250-0.0265) | 0.0177 (0.0176-0.0178) | 0.0212 (0.0211-0.0213) | 0.0136 (0.0135-0.0136) | 0.0116 (0.0115-0.0117) | 0 | 0.1701 (0.1696-0.1707) |
| **YRI** | 0.1605 (0.1599-0.1611) | 0.1725 (0.1718-0.1730) | 0.1522 (0.1517-0.1526) | 0.1747 (0.1741-0.1752) | 0.1731 (0.1725-0.1737) | 0.1738 (0.1706-0.1771) | 0.1756 (0.1751-0.1762) | 0.1715 (0.1709-0.1720) | 0.1757 (0.1752-0.1763) | 0.1598 (0.1593-0.1604) | 0.1701 (0.1696-0.1707) | 0 |

Note: The numbers in parentheses are 95% confidence intervals for each pairwise F_ST_.
