# Supplementary material for: Genetic structure, divergence and admixture of Han Chinese, Japanese and Korean populations
Source: Hereditas. 2018 Apr 6;155:19. doi: 10.1186/s41065-018-0057-5 (PMC5889524; doi:10.1186/s41065-018-0057-5)
Supplement: Supplementary file 3 — Table S1. Genotype data used in this research. (DOCX 21 kb) [file 41065_2018_57_MOESM3_ESM.docx]

**Table S1 | Genotype data used in this research.**

| **Label** | **Population** | **Sample Size (After QC)** | **Markers** | **Data Source** |
| --- | --- | --- | --- | --- |
| BMON | Buryat Mongolian | 25 | 859,841 | Xing, J., et al. (2013). |
| CDX | Chinese Dai in Xishuangbanna | 67 | 1,186,118 | 1000G |
| CEU | Northern Europeans from Utah | 177 | 928,655 | HapMap |
| CHB | North Han Chinese | 89 | 928,655 | HapMap |
| CHS | South Han Chinese | 93 | 884,233 | 1000G |
| JPRK | Ryukyuan | 49 | 58,528 | PanAsia |
| JPT | Japanese in Tokyo | 90 | 928,655 | HapMap |
| KHV | Kinh in Ho Chi Minh City, Vietnam | 44 | 1,186,114 | 1000G |
| KOR | South Korean | 100 | 934,968 | Affymetrix6.0 |
| QHM | Deedu (DU) Mongolians in Qinghai-Tibetan Plateau | 4 | 859,841 | Xing, J., et al. (2013). |
| TIB | Tibetan | 46 | 934,968 | Affymetrix6.0 |
| YRI | Yoruba in Ibadan, Nigeria | 180 | 928,655 | HapMap |

Note: This table lists the whole genome dataset we used in this work. These SNPs data have several sources. Six populations (CEU, CHB, JPT, KOR, TIB and YRI), which have the densest SNPs, are from HapMap Project[[1](file:///D:\王宇辰\Project\%5b20140821%5dEA%20Manuscript\%5b09初稿%5d\Supplementary_Tables.docx#_ENREF_1)] or provided by our research partner (both Affymetrix 6.0 microarray data). Chinese Dai in Xishuangbanna (CDX), South Han Chinese (CHS) and Vietnamese Kinh in Ho Chi Minh City (KHV) are provided by 1000 Genome Project[[2](file:///D:\王宇辰\Project\%5b20140821%5dEA%20Manuscript\%5b09初稿%5d\Supplementary_Tables.docx#_ENREF_2)]. A recent published work studying high-altitude Mongolians, providing DU Mongolian collected in Qinghai (QHM) and Buryat Mongolian (BMON). PanAsia SNP Project[[3](file:///D:\王宇辰\Project\%5b20140821%5dEA%20Manuscript\%5b09初稿%5d\Supplementary_Tables.docx#_ENREF_3)], despite with low density SNPs, uniquely provides Ryukyuan population(JPRK). The geographic distribution of these samples can be found in Figure 1A.
