# Supplementary material for: Genetic structure, divergence and admixture of Han Chinese, Japanese and Korean populations
Source: Hereditas. 2018 Apr 6;155:19. doi: 10.1186/s41065-018-0057-5 (PMC5889524; doi:10.1186/s41065-018-0057-5)
Supplement: Supplementary file 12 — Table S3. Part of F3 test results. (DOCX 18 kb) [file 41065_2018_57_MOESM12_ESM.docx]

**Table S3** **| Part of F_3_ test results.**

| **Source 1** | **Source 2** | **Target** | **F_3_ Value** | **Standard error** | **Z score** |
| --- | --- | --- | --- | --- | --- |
| CEU | CHB | BMON | -0.00537 | 0.000649 | -8.285 |
| CEU | CHS | BMON | -0.00473 | 0.000674 | -7.012 |
| CEU | JPT | BMON | -0.00635 | 0.000661 | -9.611 |
| CEU | JPRK | BMON | -0.00279 | 0.0008 | -3.483 |
| CEU | KOR | BMON | -0.00781 | 0.000623 | -12.545 |
| BMON | CDX | CHB | -0.00153 | 0.000265 | -5.784 |
| BMON | CHS | CHB | -0.00127 | 0.000157 | -8.116 |
| BMON | KHV | CHB | -0.00123 | 0.000248 | -4.952 |
| CDX | JPT | CHB | -0.00064 | 0.000189 | -3.401 |
| CDX | KOR | CHB | -0.00116 | 0.000143 | -8.132 |
| CDX | QHM | CHB | -0.0015 | 0.000223 | -6.729 |
| CDX | TIB | CHB | -0.0019 | 0.000237 | -8.052 |
| CHS | JPT | CHB | -0.00041 | 0.000115 | -3.581 |
| CHS | KOR | CHB | -0.00046 | 0.000089 | -5.191 |
| CHS | QHM | CHB | -0.00136 | 0.000137 | -9.971 |
| CHS | TIB | CHB | -0.00131 | 0.000146 | -8.971 |
| JPT | KHV | CHB | -0.00051 | 0.000167 | -3.073 |
| KHV | KOR | CHB | -0.00098 | 0.000133 | -7.401 |
| KHV | QHM | CHB | -0.00117 | 0.000203 | -5.787 |
| KHV | TIB | CHB | -0.00182 | 0.000208 | -8.727 |
| BMON | CDX | CHS | -0.00157 | 0.000226 | -6.965 |
| BMON | KHV | CHS | -0.00082 | 0.000233 | -3.51 |
| CDX | CHB | CHS | -0.00131 | 0.000115 | -11.436 |
| CDX | JPT | CHS | -0.00154 | 0.000167 | -9.246 |
| CDX | JPRK | CHS | -0.00087 | 0.000222 | -3.943 |
| CDX | KOR | CHS | -0.00201 | 0.000135 | -14.918 |
| CDX | QHM | CHS | -0.00145 | 0.000203 | -7.16 |
| CDX | TIB | CHS | -0.00191 | 0.000209 | -9.142 |
| CHB | KHV | CHS | -0.00086 | 0.000104 | -8.306 |
| JPT | KHV | CHS | -0.00096 | 0.000147 | -6.566 |
| KHV | KOR | CHS | -0.00139 | 0.000122 | -11.353 |
| KHV | QHM | CHS | -0.00068 | 0.0002 | -3.382 |
| KHV | TIB | CHS | -0.00137 | 0.000201 | -6.8 |
| BMON | JPRK | JPT | -0.00138 | 0.000244 | -5.639 |
| CDX | JPRK | JPT | -0.00132 | 0.000229 | -5.756 |
| CHB | JPRK | JPT | -0.00205 | 0.000166 | -12.344 |
| CHS | JPRK | JPT | -0.00199 | 0.000176 | -11.312 |
| JPRK | KHV | JPT | -0.00155 | 0.000209 | -7.404 |
| JPRK | KOR | JPT | -0.0021 | 0.000139 | -15.168 |
| JPRK | QHM | JPT | -0.00115 | 0.000215 | -5.349 |
| JPRK | TIB | JPT | -0.00159 | 0.000231 | -6.896 |
| CHB | JPT | KOR | -0.00061 | 0.000098 | -6.258 |
| CHB | JPRK | KOR | -0.00056 | 0.000154 | -3.604 |
| CHS | JPT | KOR | -0.00056 | 0.000106 | -5.317 |
| CHS | JPRK | KOR | -0.00045 | 0.000167 | -2.663 |
| CDX | CEU | QHM | -0.00298 | 0.000679 | -4.385 |
| CEU | CHB | QHM | -0.00793 | 0.000547 | -14.508 |
| CEU | CHS | QHM | -0.0072 | 0.000587 | -12.256 |
| CEU | JPT | QHM | -0.00803 | 0.000579 | -13.876 |
| CEU | JPRK | QHM | -0.0047 | 0.000706 | -6.652 |
| CEU | KHV | QHM | -0.00277 | 0.000672 | -4.117 |
| CEU | KOR | QHM | -0.00968 | 0.000538 | -17.968 |
| CEU | TIB | QHM | -0.00481 | 0.000561 | -8.57 |
| CHB | YRI | QHM | -0.0022 | 0.000688 | -3.199 |
| KOR | YRI | QHM | -0.00315 | 0.000703 | -4.473 |

Note: The groups with significant negative F_3_ value (Z≤-2.58，p≤0.01) are reserved.
